# Novel (Phenothiazinyl)Vinyl-Pyridinium Dyes and Their Potential Applications as Cellular Staining Agents

**DOI:** 10.3390/ijms22062985

**Published:** 2021-03-15

**Authors:** Bianca Stoean, Dumitrita Rugina, Monica Focsan, Ana-Maria Craciun, Mǎdǎlina Nistor, Tamas Lovasz, Alexandru Turza, Ioan-Dan Porumb, Emese Gál, Castelia Cristea, Luminita Silaghi-Dumitrescu, Simion Astilean, Luiza Ioana Gaina

**Affiliations:** 1 Research Center on Fundamental and Applied Heterochemistry, Faculty of Chemistry and Chemical Engineering, Babeş-Bolyai University, 11 Arany Janos Street, 400028 Cluj-Napoca, Romania; sba2488@chem.ubbcluj.ro (B.S.); tlovasz@chem.ubbcluj.ro (T.L.); pdan@chem.ubbcluj.ro (I.-D.P.); emese@chem.ubbcluj.ro (E.G.); castelia@chem.ubbcluj.ro (C.C.); lusi@chem.ubbcluj.ro (L.S.-D.); 2 Biochemistry Department, Faculty of Veterinary Medicine, University of Agricultural Science and Veterinary Medicine, 3-5 Calea Manastur Street, 400327 Cluj-Napoca, Romania; oliviapreda@gmail.com (D.R.); nistor.madalina@usamvcluj.ro (M.N.); 3 Nanobiophotonics and Laser Microspectroscopy Center, Interdisciplinary Research Institute in Bio-Nano-Sciences, Babes-Bolyai University, 42 Treboniu Laurian Street, 400271 Cluj-Napoca, Romania; monica.iosin@phys.ubbcluj.ro (M.F.); ana.gabudean@ubbcluj.ro (A.-M.C.); simion.astilean@phys.ubbcluj.ro (S.A.); 4 Department of Mass Spectrometry, Chromatography and Applied Physics, National Institute for Research and Development of Isotopic and Molecular Technologies, 67-103 Donat Street, 400293 Cluj-Napoca, Romania; alexandru.turza@itim-cj.ro; 5 Biomolecular Physics Department, Faculty of Physics, Babeş-Bolyai University, 1 M. Kogalniceanu Street, 400084 Cluj-Napoca, Romania

**Keywords:** (phenothiazinyl)vinyl-pyridinium (PVP) dyes, sonochemistry, cellular staining, melanoma cells, epi-fluorescence imaging, two-photon excited fluorescence lifetime imaging

## Abstract

We report here the synthesis and structural characterization of novel cationic (phenothiazinyl)vinyl-pyridinium (PVP) dyes, together with optical (absorption/emission) properties and their potential applicability as fluorescent labels. Convective heating, ultrasound irradiation and mechanochemical synthesis were considered as alternative synthetic methodologies proficient for overcoming drawbacks such as long reaction time, nonsatisfactory yields or solvent requirements in the synthesis of novel dye (E)-1-(3-chloropropyl)-4-(2-(10-methyl-10H-phenothiazin-3-yl)vinyl)pyridin-1-ium bromide **3d** and its *N*-alkyl-2-methylpyridinium precursor **1c**. The *trans* geometry of the newly synthesized (E)-4-(2-(7-bromo-10-ethyl-10H-phenothiazin-3-yl)vinyl)-1-methylpyridin-1-ium iodide **3b** and (E)-1-methyl-4-(2-(10-methyl-10H-phenothiazin-3-yl)vinyl)pyridin-1-ium tetrafluoroborate **3a′** was confirmed by single crystal X-ray diffraction. A negative solvatochromism of the dyes in polar solvents was highlighted by UV-Vis spectroscopy and explanatory insights were supported by molecular modeling which suggested a better stabilization of the lowest unoccupied molecular orbitals (LUMO). The photostability of the dye **3b** was investigated by irradiation at 365 nm in different solvents, while the steady-state and time-resolved fluorescence properties of dye **3b** and **3a′** in solid state were evaluated under one-photon excitation at 485 nm. The in vitro cytotoxicity of the new PVP dyes on B16-F10 melanoma cells was evaluated by WST-1 assay, while their intracellular localization was assessed by epi-fluorescence conventional microscopy imaging as well as one- and two-photon excited confocal fluorescence lifetime imaging microscopy (FLIM). PVP dyes displayed low cytotoxicity, good internalization inside melanoma cells and intense fluorescence emission inside the B16-F10 murine melanoma cells, making them suitable staining agents for imaging applications.

## 1. Introduction

In the last years, the methine dyes gained special attention in bioimaging due to their high molar extinction coefficients, high fluorescence quantum yields, large range of absorption and emission wavelengths reaching near infrared (NIR) domain and a good ability to penetrate through the cell membranes. The applications of methine dyes in the cell imaging are based on their selective accumulation in the target cellular organelles, cytoskeletal components, DNA, RNA G-quadruplexes [1], nucleotides, neurotransmitters, and many other biological macromolecules.

Fluorescent probes, particularly emitting in NIR, based on polymethine dyes [2], polyfluorinated cyanine dyes [3], and cyanine-benzothiazole hybrid system [4] revealed high selectivity towards mitochondria. The lysosomal compartment of different cell lines such as HF-P4, BLM, U-2 OS and A-2058 were selectivity labeled by fluorescent Coumarin Troger’s base derivatives with cyanine substituents [5]. The selective staining of the cytoplasm was achieved by using different classes of organic dyes. For example, Schiff bases-boron complexes were successfully used for the in vitro cytoplasmic staining in B16-F10 murine melanoma cells [6], while fluorophores containing thiophene moieties were employed in the selective staining of cytoplasm in live mouse embryonic fibroblasts [7].

Other methine derivatives such us triphenylmethane Brilliant Blue G [8] and styryl dye LDS 820 [9], respectively, can be used as fluorescent NIR fluorescent labels for amyloid fibrils, which are associated with numerous neurodegenerative diseases. A large number of commercially available or newly synthesized styryl dyes are currently in use or under investigation for their ability to act as staining agents in cell and tissue imaging [10].

Continuing our previous investigations dedicated to the synthesis and characterization of (phenothiazinyl)vinyl-pyridinium (PVP) dyes [11], in this work the series of cationic methine dyes was extended with three new representatives (i.e., **3b**, **3a′** and **3d**), exhibiting structural adjustments designed to modulate their optical properties by introducing an auxochromic bromo-substituent at the electron-donor phenothiazine unit **3b** and to modify the aggregation properties of the dye by attaching different alkyl chains and varying the counter ion at the pyridinium electron withdrawing unit **3a′**, **3d**. In our pursuit for finding greener synthetic alternatives to the traditional methods to carry out organic synthesis, in our previous experiments the PVP dyes were conveniently synthesized by microwaves assisted condensation of phenothiazine carbaldehyde with methylpyridinium salts in dry media, while in this work we examine the benefits of ultrasound assisted and mechanochemical synthetic procedures, as candidates for environmentally friendly protocols proficient for overcoming drawbacks such as long reaction time, nonsatisfactory yields or solvent requirements. Based on the documented potential of methine dyes for practical applications as fluorescent markers the photophysical behavior of PVP dyes in biological environment (i.e., B16-F10 murine melanoma cells) was investigated via fluorescence imaging, as part of our search for novel cellular staining agents.

## 2. Results and Discussion

### 2.1. Synthesis of PVP

**Scheme 1:**
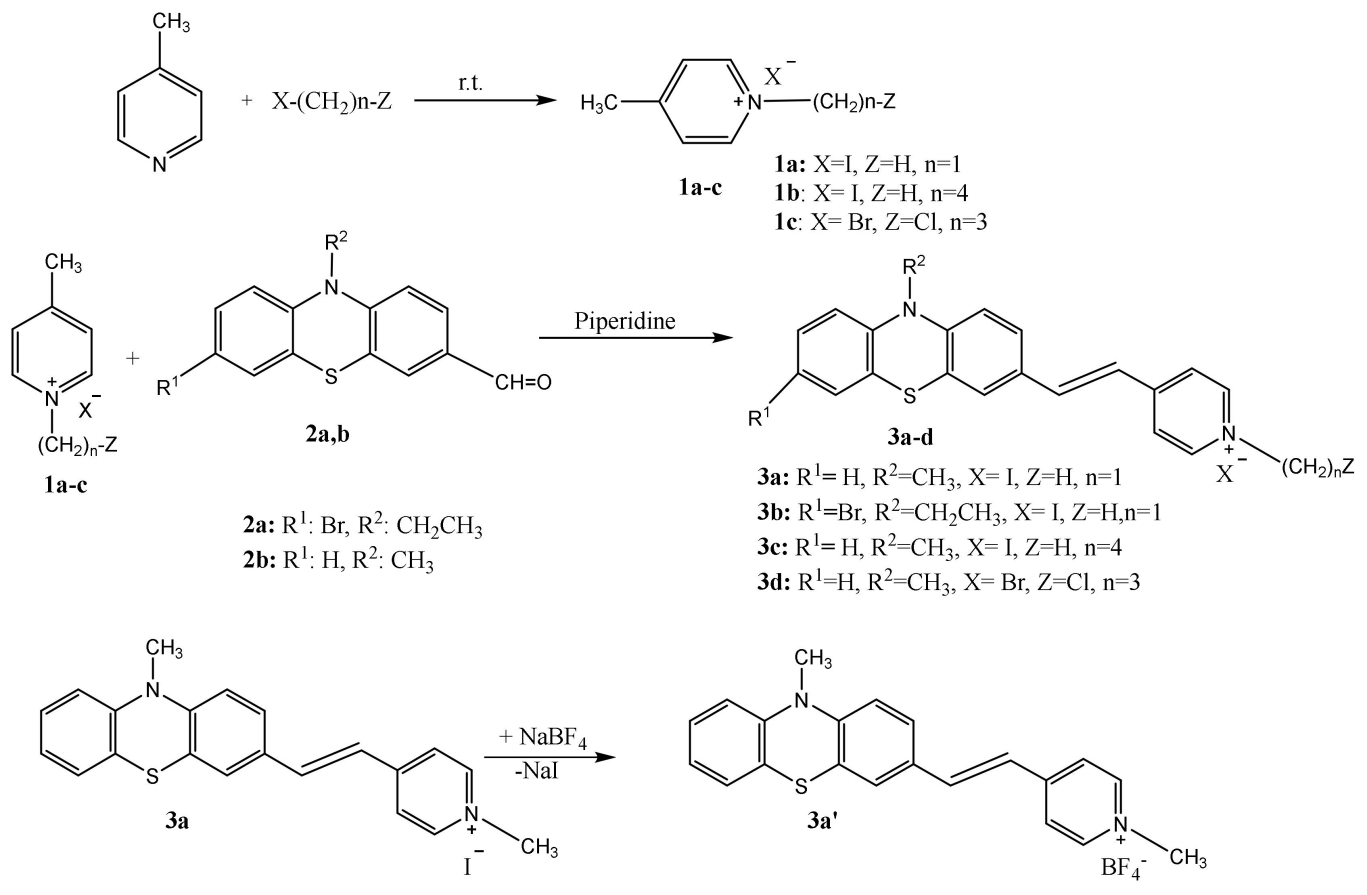
The synthesis of (phenothiazinyl)vinyl-pyridinium (PVP) dyes and their precursors.

The synthetic path presented in Scheme 1 contains two reaction steps, the preparation of the starting pyridinium salts **1a**–**c** by the alkylation of 4-picoline with halogenated alkyl derivatives, followed by the preparation of PVP dyes **3a**–**d** by Knoevenagel condensation of phenothiazine-carbaldehydes with the alkyl pyridinium salts.

Different strategies have been addressed for the optimization of the reaction conditions. Thus, the alkylation of picoline was achieved in homogeneous media using propanol solution under stirring or ultrasound irradiation as well as under solvent free conditions, to produce 1-(3-chloropropyl)-4-methylpyridin-1-ium bromide **1c** (in 80% yield by heating in homogeneous solution, 86% yield under ultrasound irradiation and 68% yield under solvent free mechanical stirring). For the synthesis of PVP dye **3d** convective heating (yield 70%), ultrasound irradiation (yield 80%) and solvent free mechanochemical procedures (yield 50%) were also applied, the higher reaction yields being achieved by ultrasound irradiation of the reaction mixture. A comparison between the outcomes of the use of traditional convective heating with nonconventional alternative energy sources such as ultrasound and mechanical mixing emphasizes the superiority of the methods using homogeneous reaction media for the condensation reaction. The lowest yields obtained by applying the mechanical stirring method are explicable taking into consideration that this method cannot benefit from the improved heat and mass transfer due to high diffusion rates in low viscosity medium ensured by the use of a solvent. Benefitting from a better energy efficiency, the sonochemical method gave superior results in terms of product yield and reaction rate as compared to convective heating procedure.

The structures of PVP dyes were assigned based on high resolution NMR spectra (ESI Figures S1–S4). In the ^1^H NMR spectra of each PVP dyes **3a**–**d**, **3a′** the signals of the vinyl protons appeared highly deshielded and split in doublets with vicinal coupling constants ^3^J 15.6 -16.5 Hz, pointing towards geometrical *trans*-isomers, in agreement with the stereochemistry of the Knoevenagel condensation which produce the thermodynamically stable *E*-alkenes. The same geometry was found in solid state by single crystal X-ray diffraction (XRD) for dyes **3a′** and **3b**.

### 2.2. X-ray Crystallographic Data

Crystal and molecular structure of dyes **3a′** and **3b** were determined by single crystal XRD. The compounds crystallize in the centrosymmetric P2_1_/c space group of monoclinic crystal system.

**Figure 1:**
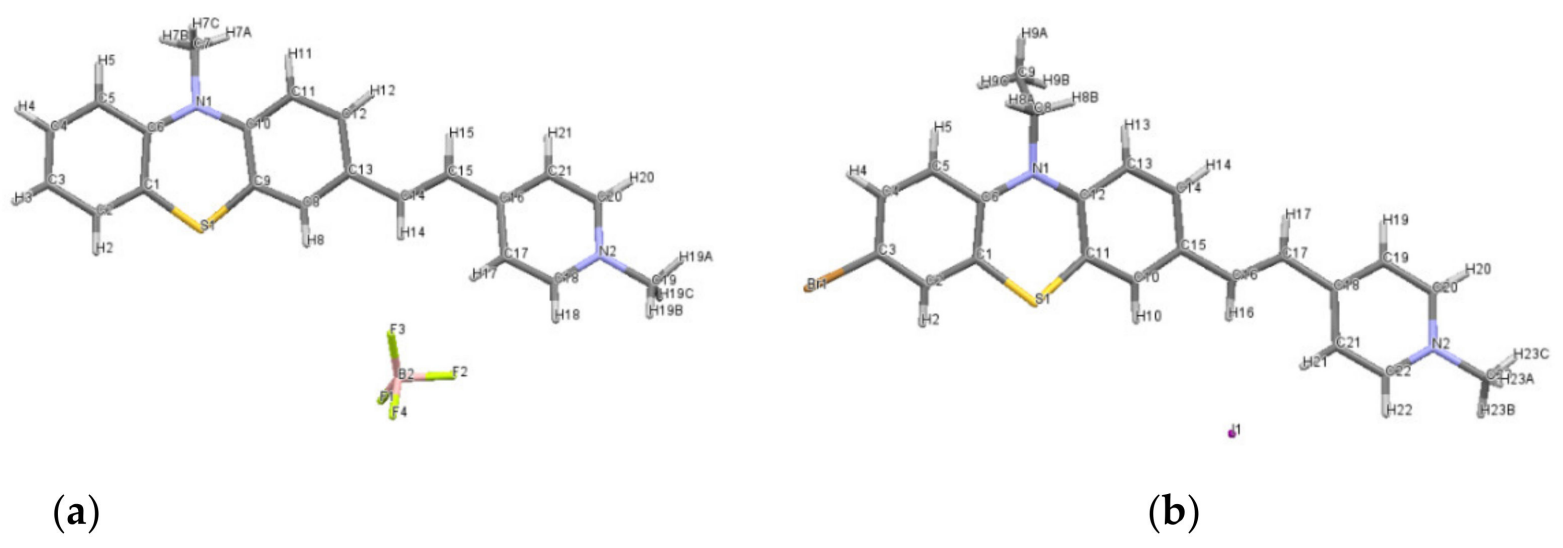
Molecular structure plots for: (**a**) PVP **3a′** with tetrafluoroborate counter anion, (**b**) PVP **3b** with iodide counter anion.

The molecular structure for the dyes **3a′** and **3b** is illustrated in Figure 1 which shows that the asymmetric unit consists of one phenothiazinyl vinyl-pyridinium cation and one counter anion: tetrafluoroborate (BF_4_^−^) in **3a′** or iodide in **3b**, respectively.

The *E* configuration of the double bond linking the phenothiazine and pyridinium units which was assumed based on NMR data recorded in solution, was confirmed in the solid state by XRD on crystals. The length of the vinyl double bond is 1.328 Å in **3a′**, and 1.338 Å in **3b**, respectively, both shorter than the reported value for dye **3c** (1.343 Å [11]). The folding angle between the aromatic rings of the phenothiazine unit displayed by **3a′** was 179.45° and 170.13° for **3b**, respectively, higher values as compared to **3c** (146.9° [11]), suggesting that the phenothiazine conformation was shaped towards a planar geometry; these structural features are supporting a better conjugation of the *π*-electron system in PVP dyes **3a′** and **3b**.

**Figure 2:**
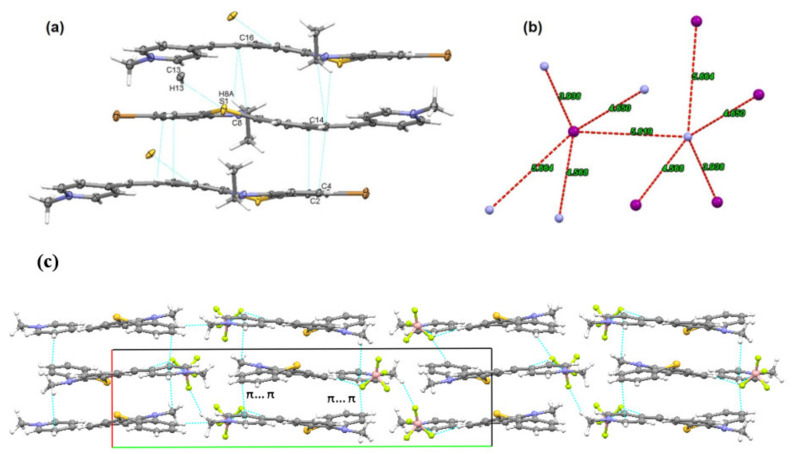
(**a**) Molecular stacking in dye **3b**; (**b**) the closest distances between I^−^ to N^+^ (nitrogen from pyridinium unit); (**c**) crystal packing of **3a′** seen along oc-*axis*.

The nature of intermolecular interactions which assure the crystal structure stability is by far driven by weak van der Waals C-H...C and π ... π interactions (Figure 2a). The molecules are found to be aggregated in distinct stackings related by a two-fold screw axis, the angle between these stacking being 53.60°. The molecules in each stack are parallel to each other, but there are two values recorded for the distances between the planes passing through two adjacent molecules: one distance is 3.537 Å demonstrating π ... π interactions (C4 ... C16 and C14 ... C2) and the other distance is 3.452 Å, presenting C-H ... π interactions (C9-H8A ... C16). For the dye **3b**, C-H ... S interactions were shown to behave similarly to conventional hydrogen bonds, although the sulphur is mildly electronegative compared to oxygen [12].

The molecules of dye **3b** are oriented in the *oa*-direction forming chain-like arrangements, with iodide ions located in the spaces between these chains (ESI Figure S5).

Although the iodide anion (I^−^) is not found even near the pyridinium cation (N^+^), it interacts with five other N^+^ nitrogen from different neighboring molecules with distances in the range 3.938–5.664 Å (Figure 2b), these multiple electrostatic interactions contributing to the stability of the compound.

The 3D supramolecular arrangements and structural stability of the compound **3a′** are assured by a combination of C-H...F interactions between phenothiazine and pyridinium cation to tetrafluoroborate anion and π... π interactions between the phenyl ring of phenothizine to aromatic pyridinium fragment (Figure 2c). Donor to acceptor H...F contact limits are characterized by moderate distances (2.459–2.610 Å), meanwhile the C-H...F angles are situated between 126.65° and 170.56°.

### 2.3. Optical Properties

From Table 1 summarizing the structural characteristics and spectral data recorded for PVP dyes **3a–d** and **3a′**, it can be seen that the position of the absorption maxima was not affected by neither counter ion (iodide, bromide, tetrafluoroborate), alkyl moiety present in the pyridinium unit (methyl, butyl, 3-chloropropyl), nor N-functionalization of the phenothiazine unit. Changes in the electronic structure, such as auxochrome *para*-bromo-substitution of the phenothiazine heterocycle unit, did not play a major role in the absorption spectra. An absorption band located around 458 nm with high molar absorption coefficient values (ε~10^5^ M^−1^·cm^−1^) was observed for each PVP dye in agreement with possible π-π* electronic transitions. PVP dyes **3a**–**d** do not show fluorescence emission in organic or aqueous solutions due to the presence of the halide counter ion susceptible of photo-inducing the electron transfer to the dye’s LUMO, but show emission in solid state (see discussion below and Figure 7). If the counter ion was changed in **3a′** with trifluoroborate, this compound displayed emission both in solid state (720 nm) and solution (590 nm in water, 606 nm in MeOH, 605 nm in DMSO and acetone, respective 708 nm in chloroform). A hypsochromic shift of the fluorescence emission for dye **3a′** in polar protic solvents, in comparison to less polar aprotic (CHCl_3_) solvent was observed (see ESI Figure S7), this behavior appearing also in the UV-vis spectra. The dye **3a′** displays a relative low quantum yield in solution (e.g., in chloroform solution was 3.5% relative to Rhodamine 101 standard).

The two-photon excited (TPE) fluorescence spectrum of dye **3a′** in DMSO solution after excitation at 800 nm shows the emission maxima at 605 nm (see ESI Figure S8), value corresponding to the one-photon excited fluorescence in DMSO.

The chemical stability and aggregation of the PVP dyes in solution was estimated by monitoring the changes in the position of the visible absorption maximum, in different solvents, upon continuous light irradiation (λ = 365 nm) and temperature increase up to 40 °C.

**Figure 3:**
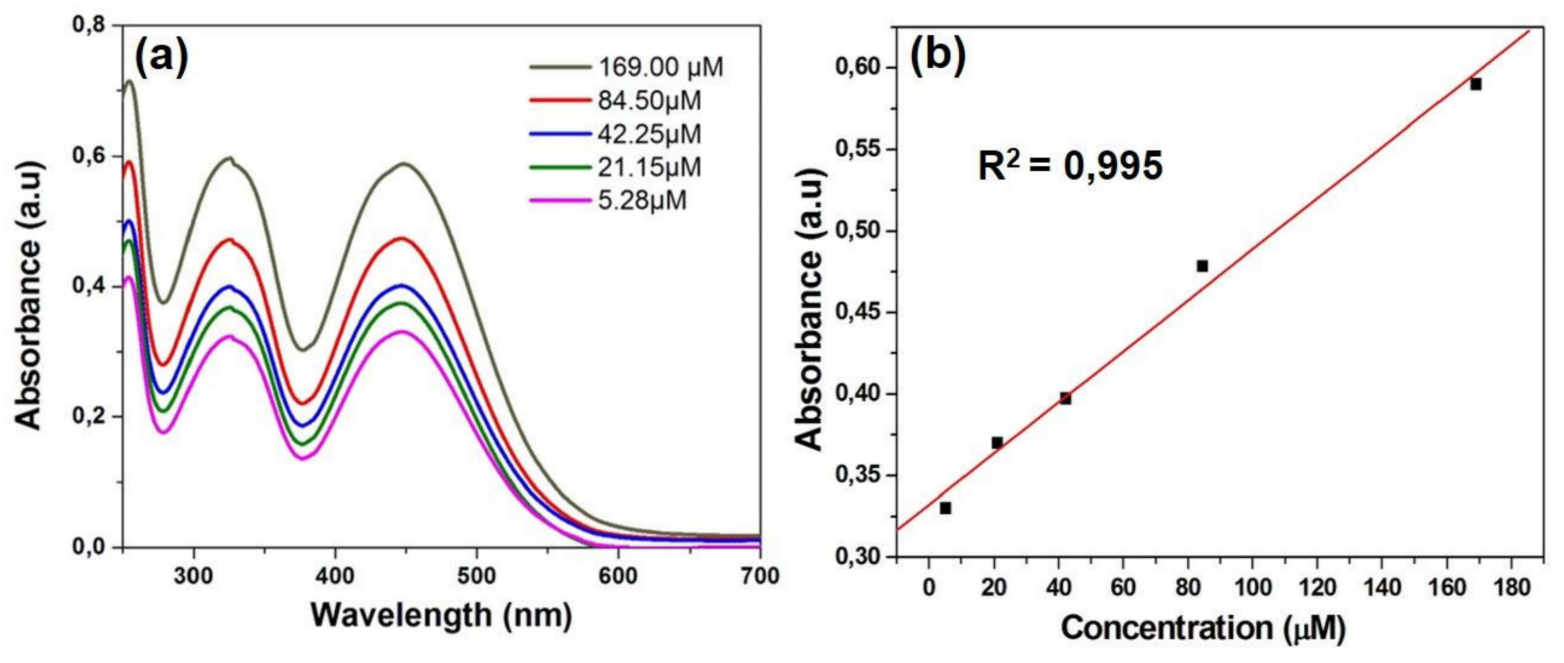
(**a**) UV-Vis absorption spectruma of dye **3b** in water at different concentrations (5.28–169 μM); (**b**) plot of absorbance maximum versus concentration.

The PVP dyes appeared to be stable in water and TRIS-EDTA buffer solution, as ESI Figure S9 shows unchanged pattern for the absorption spectrum displayed by dye **3b** in buffer solution after irradiation at 365 nm up to 90 min. It is known that in solution, methine dyes can form aggregates imparting different photophysical behavior from those observed for the monomeric species [13], but in the case of PVP in water solution there is no evidence of aggregation, as it can be seen from the UV-Vis absorption spectra of dye **3b** in water recorded at different dye concentrations (Figure 3a). The absorption pattern typical for a monomeric dye occurs in the linearity domain of Lambert Beer law, as presented in Figure 3b.

**Figure 4:**
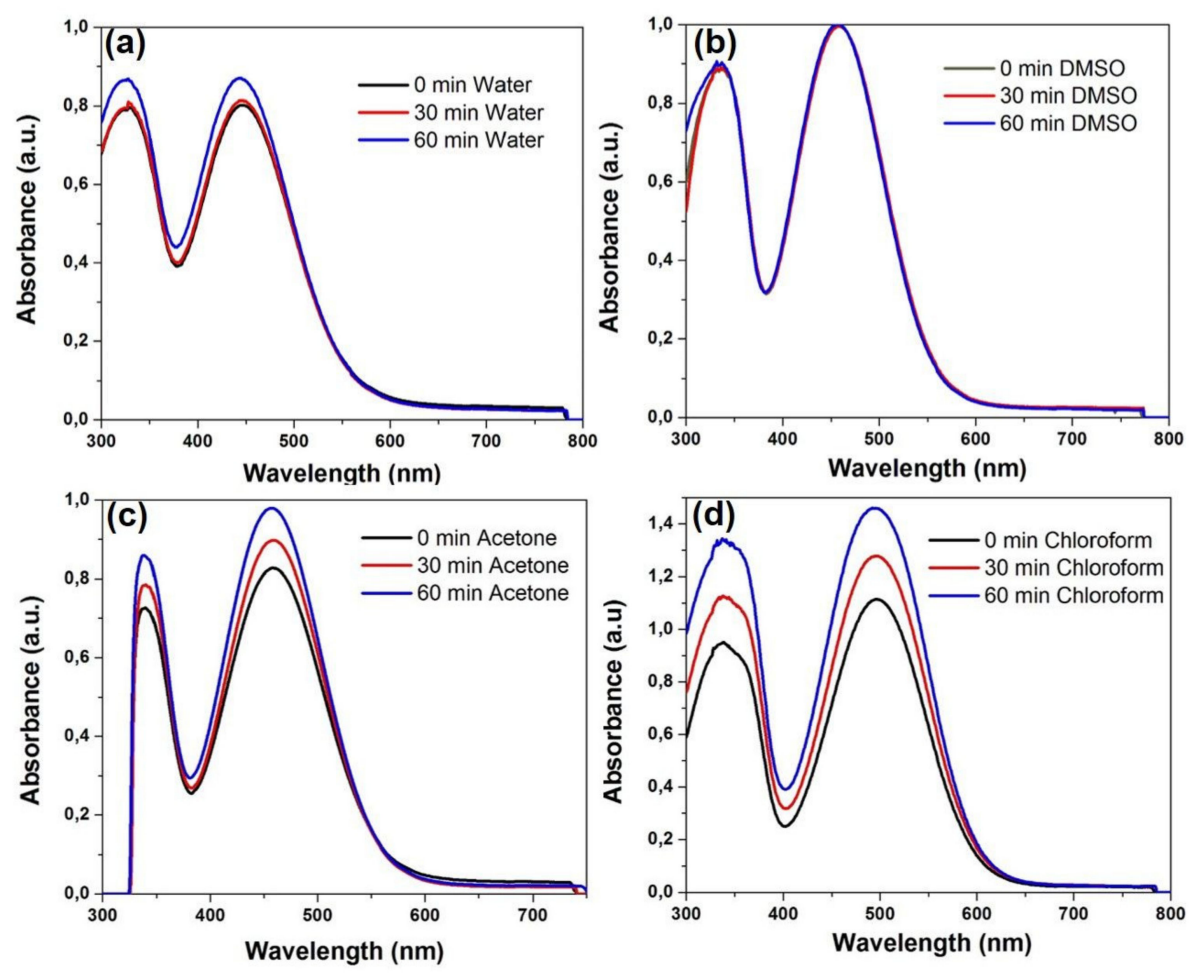
Photostability of dye **3b** under UV light irradiation (λ_ex_ = 365 nm) for 30 and 60 min in solution: (**a**) water; (**b**) DMSO; (**c**) acetone; (**d**) chloroform.

Photostability is an important parameter in bioimaging applications and for this reason the possible degradation of dye **3b** under UV light irradiation was investigated by UV-Vis spectroscopy in different solvents. Thus, solutions of **3b** in water, DMSO, acetone, chloroform, DCM and methanol, respectively, were irradiated with UV light at λ = 365 nm for 30 and 60 min, respectively, at room temperature and then the UV-Vis spectra were recorded (Figure 4 and ESI Figures S10 and S11). No degradation of dye **3b** was observed neither in water nor in organic solvent. The spectra presented reveal that the exposure of dye **3b** at UV light do not induce hydrolytic effects and the optical properties were not affected in water solution (Figure 4a). In the presence of less polar solvents (e.g., acetone, chloroform) an hyperchromic effect proportional to the increase of irradiation time was recorded, as shown in Figure 4c,d.

The hyperchromic effect, more important in chloroform and methylene chloride (Figure 4d and ESI Figure S11) may possibly be due to an aggregation of the dye in the halogenated solvents. The hyperchromic effect appeared enhanced upon heating at 40 °C (ESI Figure S12).

**Figure 5:**
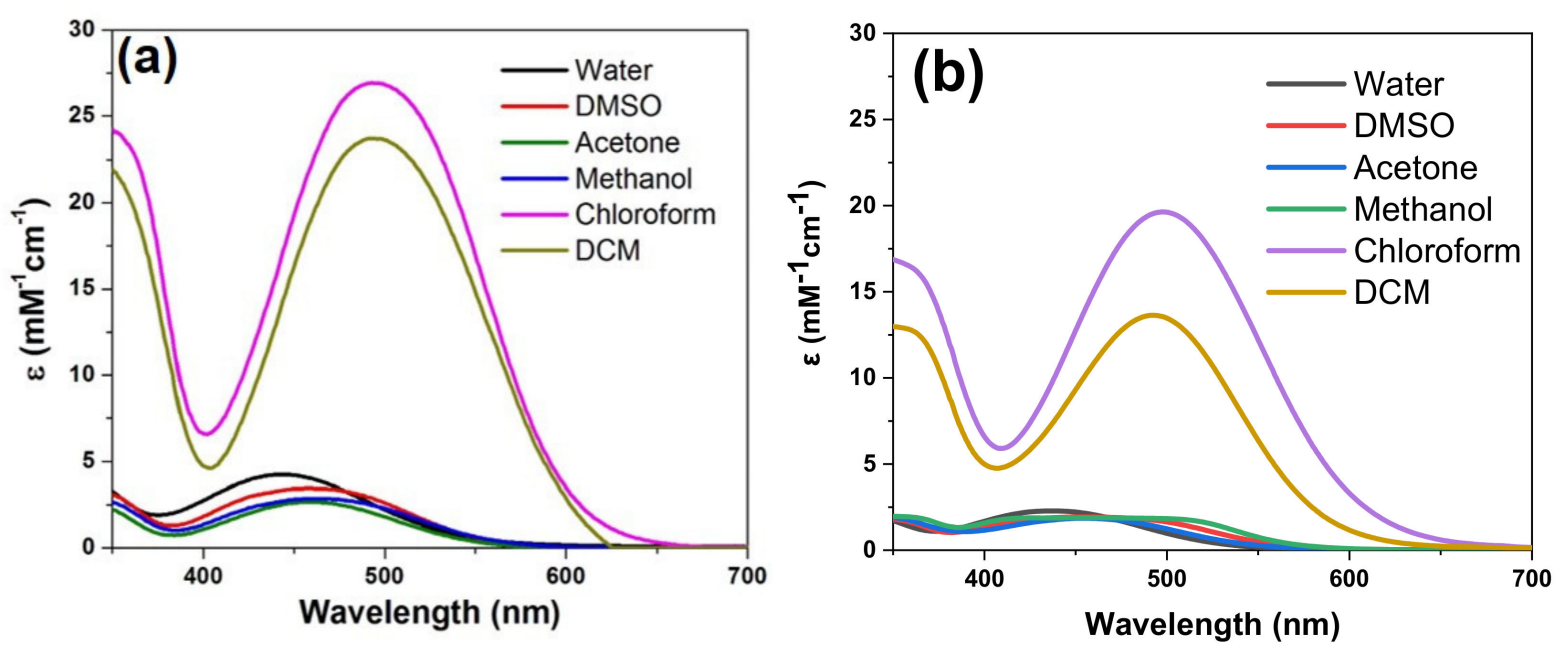
UV–Vis absorption spectra dye in different solvents: (**a**) **3b** and (**b**) **3a′**.

A hypsochromic shift of the visible absorption maxima was noticed for dye solutions in polar protic (water, methanol) or aprotic (DMSO) solvents, in comparison to less polar aprotic (CH_2_Cl_2_, CHCl_3_) solvents. The UV-Vis absorption maxima of dyes **3b**, **3d** and **3a′** in different solvents are summarized in supporting info ESI Table S1. For example, in Figure 5 is depicted the solvatochromism displayed by dyes **3b** and **3a′** in solvents of different polarities, emphasizing a red shift from 444 nm in water to 495 nm in DCM solution. The modification in counter ion, iodide in **3b** and tetrafluoroborate (BF_4_^−^) in **3a′**, does not induce changes in the UV-Vis absorption spectra.

In order to comprehend the negative solvatochromism displayed by the dyes **3a**–**d**, the experimental results were corroborated with the results of a computational approach estimating the energy gap between the frontier molecular orbitals in vacuum and in the presence of different solvents. The initial geometry optimization of the PVP dyes structure obtained by molecular mechanics calculations in Spartan’06 [14] was used in quantum calculations at semi-empirical molecular orbitals (PM3) and 6–31G(d) B3LYP DFT level of theory [15,16,17]. The generated lowest energy conformer of PVP dye was further used in the calculus of quantum molecular parameters such as E_HOMO_, E_LUMO_ and energy gap ΔE (ΔE _HOMO–LUMO_) in vacuum and methanol, DMSO, acetone, chloroform, and DCM solvents, respectively.

**Figure 6:**
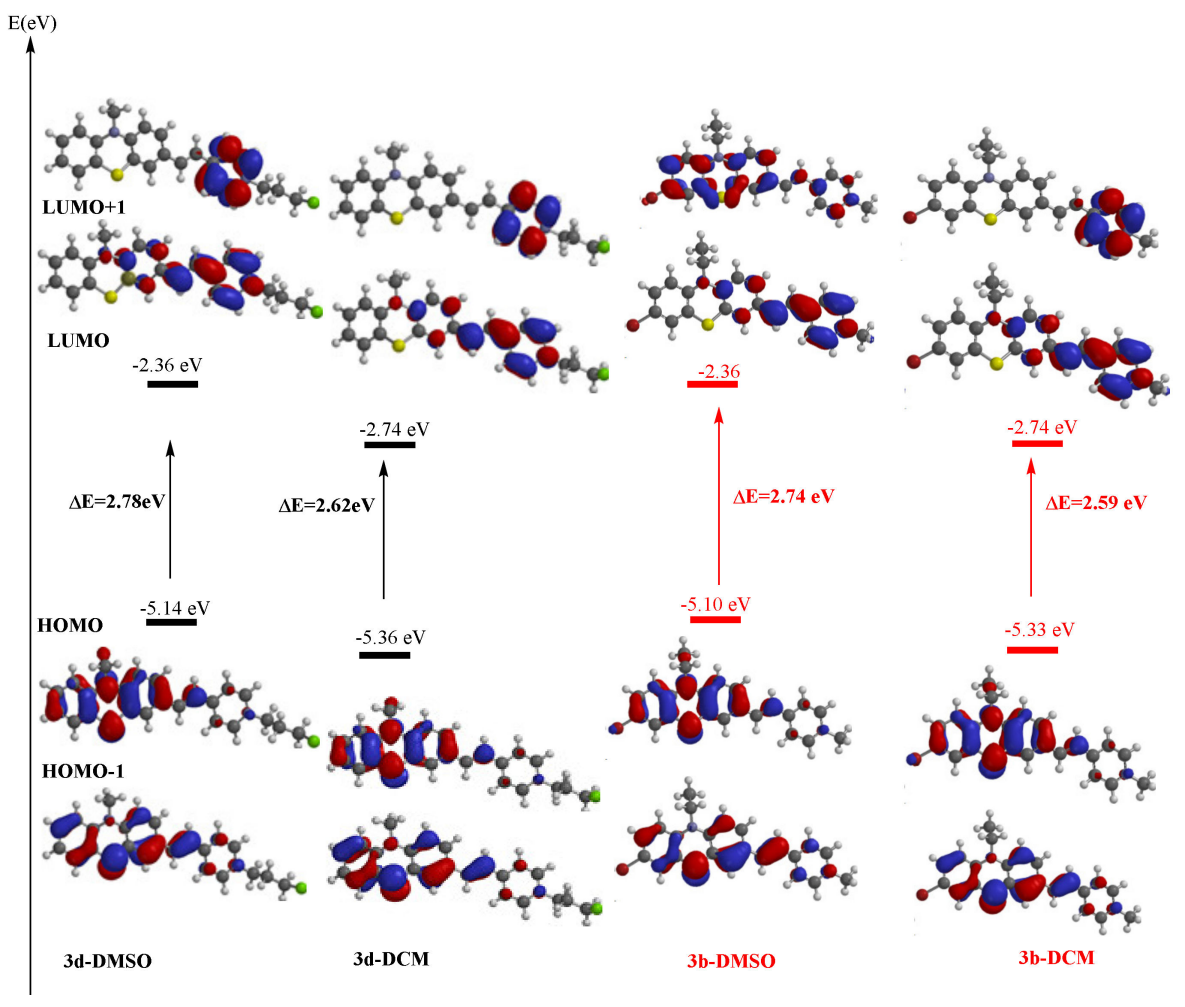
Plots of the frontier molecular orbitals and their energy in DMSO and DCM solvents for dyes **3b** and **3d** computed at DFT level of theory.

The computed electron distribution in the molecular orbitals of PVP dyes **3a**–**d** indicated occupied frontier molecular orbitals (FMO) HOMO and HOMO-1 located predominantly on the phenothiazine unit, whereas unoccupied molecular orbitals LUMO and LUMO+1 were located predominantly on the pyridine core (plots of FMO for **3a**–**d** ESI Table S2). In Figure 6 are displayed the frontier molecular orbitals plots for dyes **3b** and **3d** indicating the corresponding HOMO-LUMO energy gaps in two different solvents.

Higher values for the computed FMO energy levels were found systematically in the simulated presence of a solvent compared to vacuum conditions (ESI Table S3). The noticeable increase of the LUMO energy levels in the presence of the polar solvent DMSO is supporting the assumption of a blue shift of the absorption maximum generated by the vertical mono-electron excitation process, in total agreement with the UV-vis absorption experimental data.

Going further, we investigated the steady-state and time-resolved fluorescence emission properties of dyes **3b** and **3a′** in solid state, under one-photon excitation at 485 nm. Upon excitation at 485 nm the novel **3b** and **3a′** dyes displayed distinct fluorescence emissions in the solid state (λ_em_ = 650 nm for **3b** and λ_em_ = 720 nm for **3a′**), likewise previously reported PVP dyes **3a** and **3c** (λ_em_ = 749 nm for **3a** and λ_em_ = 668 nm for **3c** [11]).

**Figure 7:**
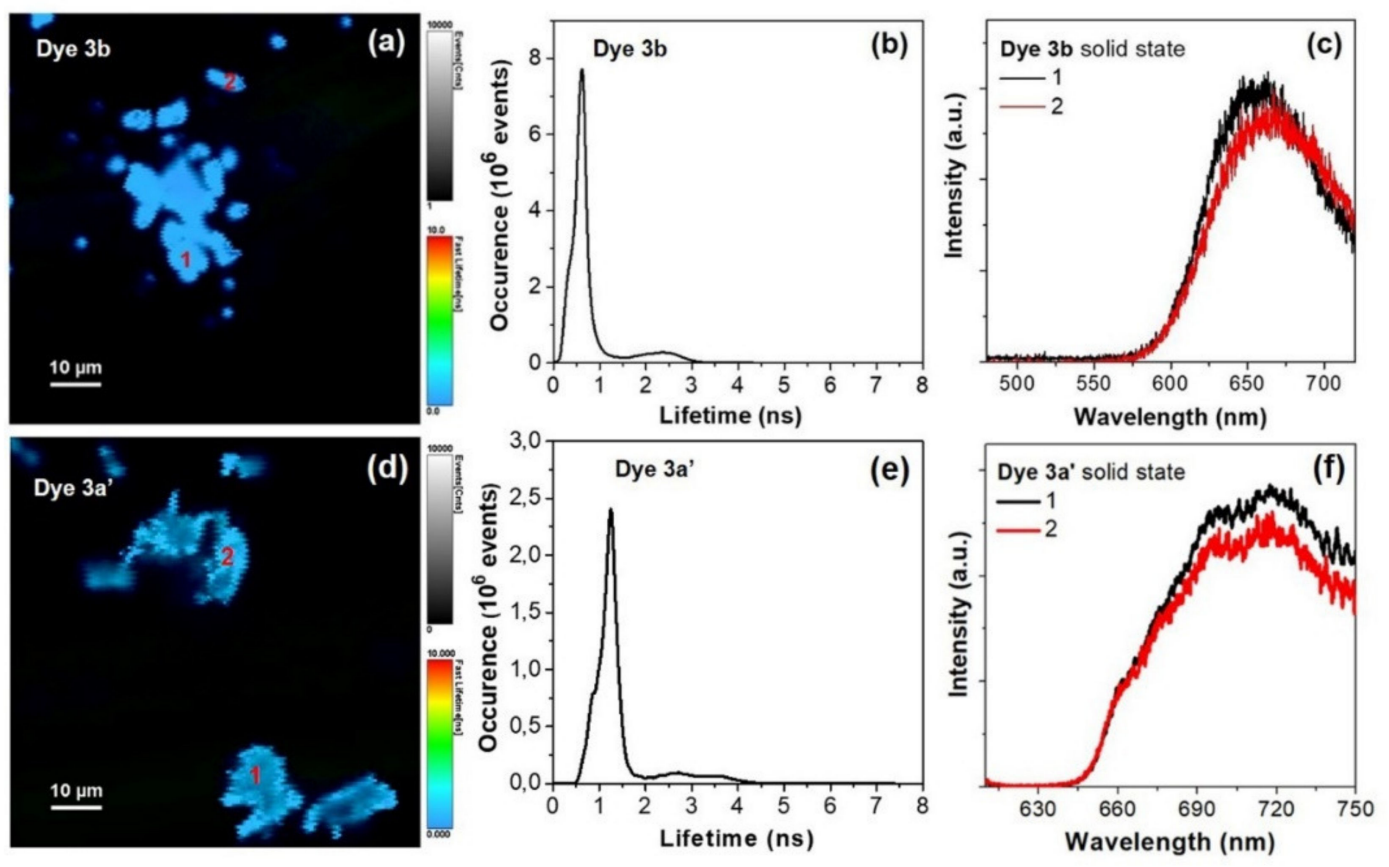
OPE-FLIM image of dyes **3b** (**a**) and **3a′** (**d**) in solid state. (**b**,**e**) Lifetime histograms corresponding to FLIM image from (**a**,**d**). (**c**,**f**) Representative one-photon excited fluorescence spectra extracted from the areas marked with 1–2 in FLIM image from (**a**,**d**). Excitation at 485 nm.

Figure 7 presents the OPE-FLIM image of dyes **3b** and **3a′** red powders deposited on microscope cover glass along with the corresponding lifetime histograms of the images and illustrative fluorescence spectra extracted from two regions of the samples.

The recorded OPE-FLIM images demonstrates that dyes **3b** and **3a′** display strong fluorescence emission in solid state, under the used excitation conditions. Moreover, in the case of dye **3b** the lifetime histogram profile of FLIM image reveals the presence of two lifetime components: one at 0.65 ns with the highest contribution and another one at 2.3 ns with a lower contribution, which could be related to the degree of intermolecular interactions occurring within the imaged aggregates which affect the excited state dynamic, considering that the fluorescence lifetime decreases when the energy transfer increases in molecular aggregates (Figure 7b). Similarly, the lifetime histogram profile of dye **3a′** reveals two lifetime components, at around 1.25 and 3 ns, also with a lower contribution (Figure 7e). Figure 7c,f presents two representative fluorescence spectra, for each of the dyes **3b** and **3a′**, extracted from the two different marked regions in the recorded OPE-FLIM images. As seen in the spectra, the one-photon excited emission of dye **3b** in solid state is localized at around 655 nm with a small variation due to above mentioned aggregation effects, while the maximum emission of dye **3a′** is localized around 720 nm.

Finally, to evaluate the potential of PVP dyes **3a**–**d** for biological applications, we assessed in vitro cytotoxicity by WST-1 assay on B16-F10 melanoma cells, while their cellular uptake and intracellular localization were analyzed by epi-fluorescence conventional imaging as well as one- and two-photon excited confocal fluorescence microscopy.

### 2.4. Staining Investigation on B16-F10 Melanoma Cells

#### 2.4.1. Cell Viability

**Figure 8:**
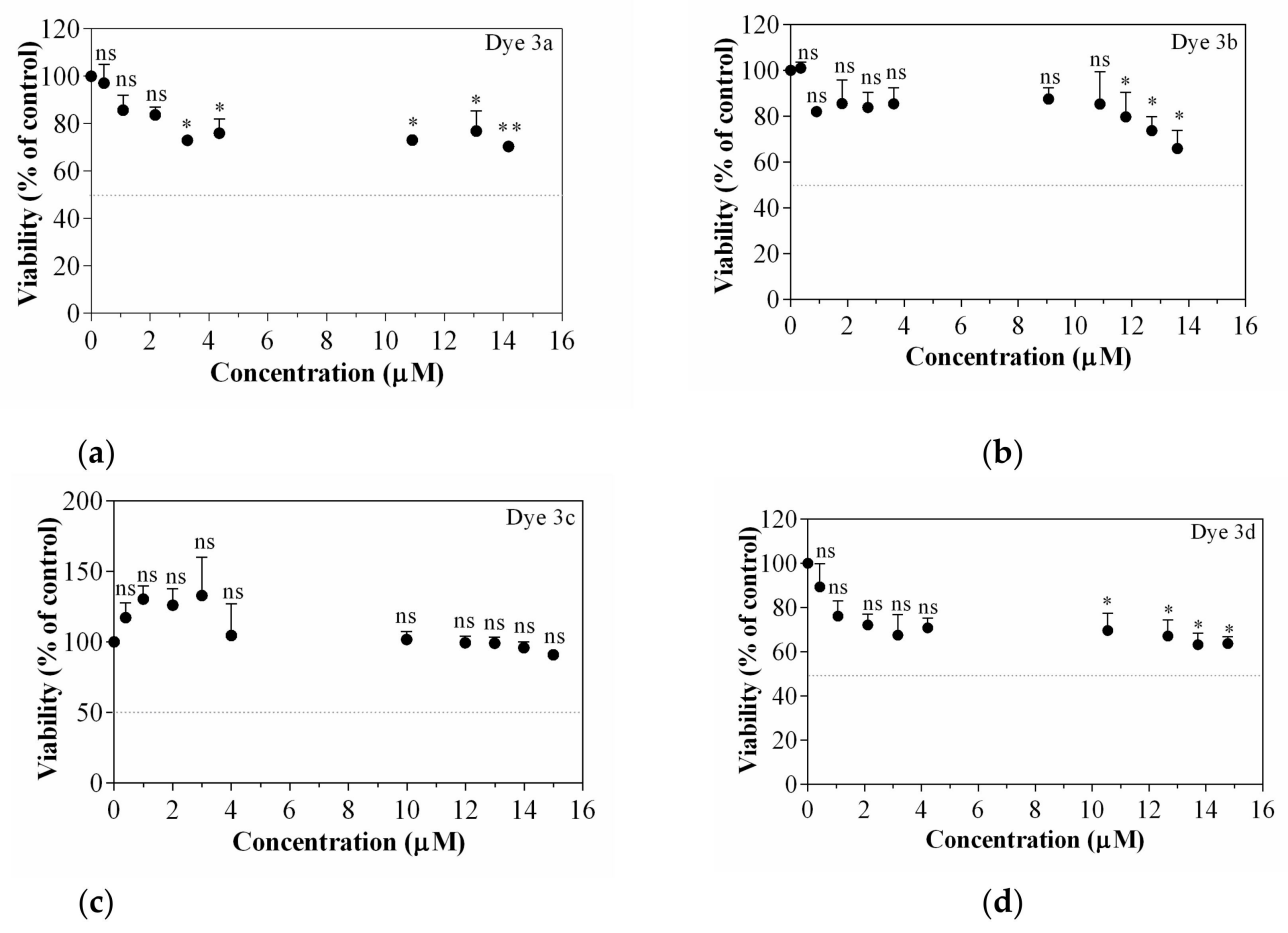
Cell viability assessed by WST-1 assay. Dyes’ influence on B16-F10 melanoma cells treated viability after 24 h of treatment, (**a**) Dye 3a; (**b**) Dye 3b; (**c**) Dye 3c; (**d**) Dye 3d. Data are expressed as mean ± SD (*n* = 3). Statistically significant difference noted as *, ns—means statistically non-significant, for a value of *p* < 0.05, compared with untreated control.

Dyes **3a**–**d** used herein to stain cells were tested on B16-F10 melanoma cells for their viability after 24 h of treatment (0–15 µM). Figure 8 attests that no dye is cytotoxic on B16-F10 cells in the concentrations range used, which qualifies them as potential molecules able to stain tumoral cells. For instance, the highest concentrations (13.7 and 14.7 µM) of dyes reduced viability with 24–30% (dye **3a**), with maximum 34% (dye **3b**), with 37% (dye **3d**) compared to untreated control. No effect on the B16-F10 cell viability was observed in dye **3d** case. In this context, for further cellular imaging assays, the concentration of 13.7 µM was selected.

#### 2.4.2. Fluorescence Imaging

**Figure 9:**
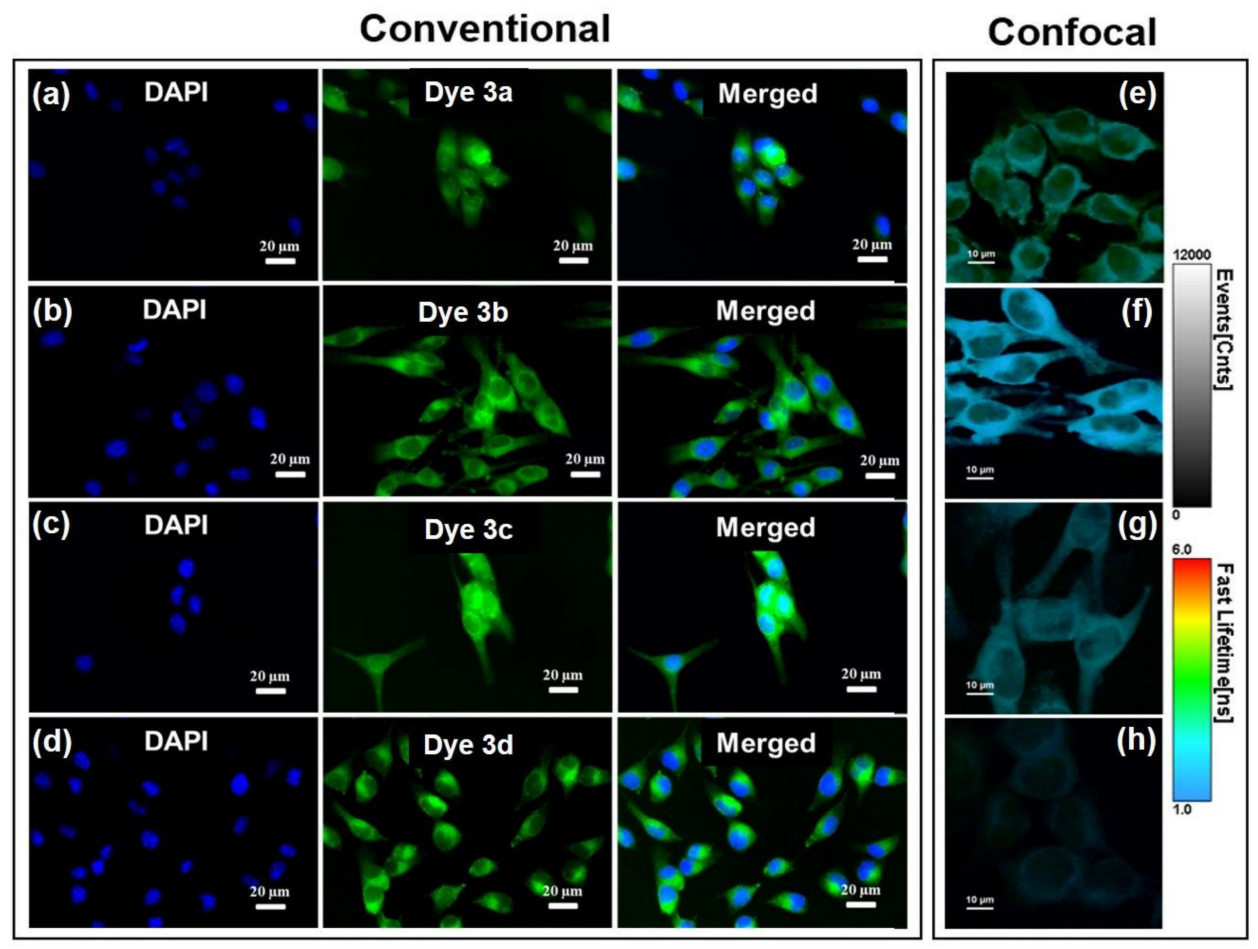
(**a**–**d**) Staining B16-F10 cells with dyes **3a**–**d** appearing colored in green in conventional epi-fluorescence imaging**.** Nuclei were stained with DAPI (blue). Excitation with blue light (450–490 nm). (**e**–**h**) Confocal OPE-FLIM images of B16-F10 melanoma cells incubated with dyes **3a**–**d.** Excitation at 485 nm.

The cellular uptake of all synthesized PVP dyes **3a**–**d** was the first step to evaluate their potential for cellular staining. After incubating B16-F10 melanoma cells with dyes for 24 h, the epi-fluorescence conventional images were acquired to evaluate their cellular uptake capability. As shown in Figure 9a–d, PVP dyes **3a**–**d** can be well-swallowed by B16-F10 melanoma cells, observing the green fluorescence in all cases, indicating consequently the good cellular uptake capability.

Specifically, Figure 9a shows that uptake of dye **3a** was uniform in the cytoplasm and nucleus by B16-F10 cells and stained both nuclei and cytoplasm of the cells as green fluorescence can be clearly observed in both regions. Considering that nucleic acids are mainly distributed in the nucleus, the collected fluorescence images led us to assume that the dye **3a** is able to simply diffuse through plasma membrane as well as nuclear membrane, interacting with these two entities (i.e., DNA/RNA). However, when compared with the following used dyes, dye **3a** is less promising for efficient cells staining and—implicitly—for the next fluorescence-based imaging applications. Dyes **3b** (Figure 9b) and **3d** (Figure 9d) can be clearly seen in entire cytoplasm, but with high tendency to accumulate in the perinuclear region. Even though experimentally determined octanol-water partition coefficients for dye **3b** comprising the hydrophobic 3-bromo-10-ethyl-phenothiazine moiety (log *p* = 0.74) and dye **3c** containing longer alkyl chain attached to the pyridinium unit (log *p* = 0.71) indicate comparable overall hydrophobicity properties (see ESI Figures S13 and S14), the existence of *n*-butyl chain seemed to favor the ability of dye **3c** to pass through the nuclear pores (Figure 9c).

It is important to note that no decrease of the fluorescence intensity was observed during recording the fluorescence imaging, concluding that dyes do not present photobleaching in the cellular environment and, consequently, are well-suited as cellular stain probes. The studied dyes appeared suitable for imagistic applications taking into consideration their good ability of light absorption spotted by their strong absorption maxima situated in the blue region of the Vis spectrum (455–460 nm with extinction coefficients ε = 10^5^ M^−1^ cm^−1^). Even though the dyes do not exhibit intense fluorescence emission in solution or solid state, the very good separation of the excitation and emission maxima (Stokes’s shifts 190–294 nm) as well as their obvious fluorescence in biological environment, are overcoming this drawback.

To better understand the localization of dyes **3a**–**d** inside the investigated cells we performed confocal imaging assays on B16-F10 cells incubated with dyes **3a**–**d**. Representative OPE-FLIM images obtained under excitation at 485 nm are shown in Figure 9e–h along with the intensity and lifetime scale bars. The results confirm that dye **3c** has the highest tendency to diffuse through the nuclear membrane and accumulate into nuclei, while dyes **3a**, **3b** and **3d** are more suitable for the staining of the cytoplasmic region, considering that only a small fluorescence signal can be detected from the nuclear region. Additionally, the employed FLIM technique offers the advantage of discriminating different staining agents based on their fluorescence lifetime. Here, the fluorescence of dyes **3a**–**d** display different lifetime inside cells, the shortest one being notably the emission of dye **3b**.

Therefore, according to the fluorescence bioimaging studies it can be mentioned that all synthesized compounds revealed their potential application as enhanced cytoplasmic staining dyes with a low cytotoxicity. Moreover, confocal OPE-FLIM assays confirm that dye **3c** has a better nuclear staining capability.

**Figure 10:**
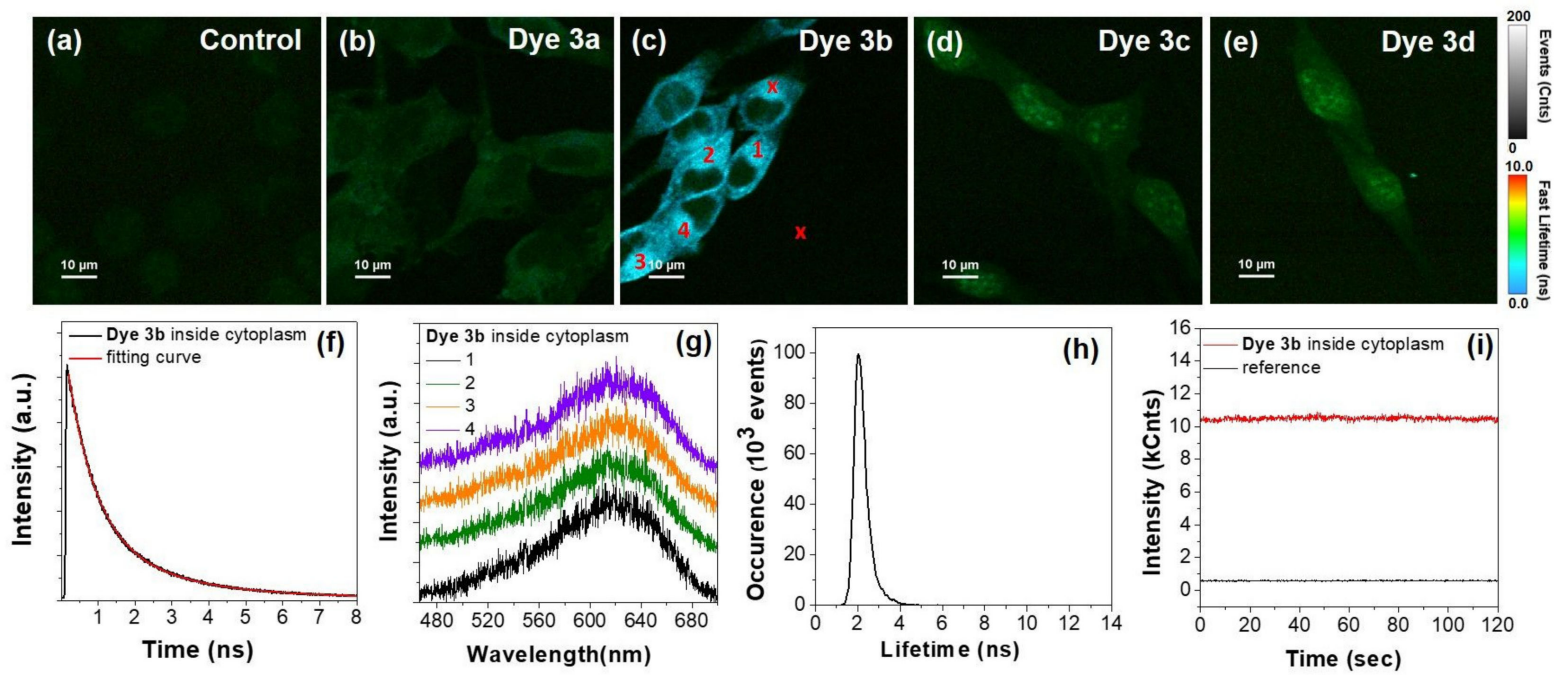
(**a**–**e**) TPE–FLIM images of control B16-F10 melanoma cells and incubated with dyes **3a**–**d**. (**f**) Lifetime decay of dye **3b** collected inside cytoplasm. (**g**) Representative TPE fluorescence spectra extracted from the areas marked with 1-4 in FLIM image from panel (**c**). (**h**) Lifetime histogram corresponding to TPE-FLIM image from (**c**). (**i**) Photostability of dye **3b** inside cytoplasm compared to reference out of cell signal, from areas marked with x symbol in panel (**c**). Excitation at 800 nm.

Further, having in mind the possibility of using the synthesized PVP dyes as NIR staining agents for TPE fluorescence imaging, a well-known noninvasive label-free and high-resolution technique, we performed imaging assays on the same B16-F10 melanoma cells incubated with the dyes **3a**–**d**, under TPE at 800 nm. Nowadays, this in vitro nonlinear bioimaging technique represents a modern and high-performance tool for relevant biological and living tissue research, a real alternative to traditional OPE, due to its unique advantages, such as improved sensitivity and axial resolution, lack of background signal and photodamage of living tissue, improved tissue penetration depth and cell viability, as well as reduced photobleaching [18]. Moreover, confocal TPE fluorescence imaging microscopy can be greatly improved when combined to FLIM approach, due to the improved sensibility and contrast that can be achieved through the mapping the fluorescence lifetime, as it is a parameter highly sensitive to environmental changes. Figure 10 presents representative TPE-FLIM images of reference melanoma B16-F10 cells (Figure 10a) and incubated with the different types of synthesized dyes **3a**–**d** (Figure 10a–e).

As shown, under the employed NIR irradiation, the control unlabeled cells exhibit a very weak TPE autofluorescence signal. In contrast, the TPE-FLIM images corresponding to cells incubated with dyes **3a**–**d** exhibit intense and traceable fluorescence signal which proves the promising ability of the as-synthesized PVP dyes to perform as reliable NIR staining agents.

After a closer examination of the images, we observed a different behavior of the investigated dyes inside cells, under TPE excitation. Specifically, we observed that the fluorescence of dye **3a** can be detected mostly within the cellular cytoplasm with a small amount inside the nuclei (Figure 10b). On the other hand, the signal of dye **3b** is localized mainly inside the cytoplasm (Figure 10c), being also the most intense from all samples, whereas dyes **3c–d** display a weak fluorescence signal within cytoplasm and much more intense in the nuclei (Figure 10d–e), in good agreement with the observation from conventional and OPE fluorescence imaging (see Figure 9). We believe that this behavior could be related to a different degree of interaction that might occur, between the employed dyes and different nuclear components. Additionally, similar to OPE-FLIM, the emission of dye **3b** displays a much shorter lifetime compared to the emission of dyes **3a**, **3c** and **3d**, which represent an additional advantage in imaging application as the interference with cellular autofluorescence (few ns) becomes lower. The fluorescence lifetime histogram extracted from the TPE-FLIM image corresponding to dye **3b** (Figure 10c) and presented in Figure 10h) reveals an average lifetime of TPE emission of 2 ns. When extracted from an area from the cytoplasm region (marked with x in Figure 10c) the TPE fluorescence lifetime decay displays a biexponential behavior. The fitting procedures reveal a lifetime of 2.3 ns (35%) and a shorter one of 0.73 ns (65%), which can be assigned to different types of molecular aggregates, in good agreement with the results from one-photon excited fluorescence imaging of dye **3b** in powder state, regarding the presence of two lifetime components (see Figure 7b).

Furthermore, we extracted several TPE fluorescence spectra from different regions in B16-F10 cells incubated with dye **3b**, that proved to the most efficient and suitable for TPE imaging. The spectra collected from the areas marked with 1–4 in TPE-FLIM image from panel c (Figure 10c) are presented in Figure 10g. All spectra share similar features while the maximum TPE fluorescence is located around 620 nm, at a lower wavelength compared to the position of the band in solid phase, under one-photon excitation (see Figure 7c). Finally, we investigated the stability of the TPE fluorescence signal of dye **3b** inside cells, under continuous irradiation. As shown in Figure 10i, the signal collected from the area marked with x in panel (c) remains stable under continuous irradiation at 800 nm over 120 s tracking time, which further confirms the ability of dye **3b** to perform as an efficient and stable staining agent for NIR imaging.

## 3. Experimental Section

### 3.1. Materials and Methods

All the materials for synthesis, reagents, and solvents were obtained from commercial suppliers and used without further purification unless otherwise noted.

Flash chromatography was performed on silica gel 60 (particle size 0.032–0.063 mm). Thin layer chromatography was performed on Merck DCAlufolien, silica gel 60 F254 and components were visualized by UV VL-4LC. The melting points were determined in capillaries with an Electrothermal 9100 instrument. Ultrasound irradiation was performed on GT SONIC-P3 Ultrasonic Cleaner Washing Equipment. NMR spectra (1D, 2D-COSY, 2D-HSQC and 2D-HMBC) were recorded at room temperature on Bruker Avance instruments (1H/13C: 400 MHz/100 MHz or 600 MHz/150 MHz) in solution (deuterated solvents (CDCl_3_ or DMSO). Elemental analyses were recorded on Thermo Scientific FlashEA™ 1112. HRMS spectra recorded on Thermo Scientific LTQ Orbitrap XL. UV-Vis absorption respective emission spectra in solvent were recorded with Perkin Elmer Lambda 35 and Perkin Elmer LS55 spectrophotometers.

### 3.2. Synthesis

The starting materials **1a**, **b** and the dyes **3a**, **c** were synthesized using the protocols reported in our previous paper [11].

1-(3-chloropropyl)-4-methylpyridin-1-ium bromide **1c**

A. Classical procedure:

In a round-bottom flask, 4-methylpyridine (0.051 mol, 5 mL) and 1-bromo-3-chloropropane (0.05 mmol, 5.08 mL) in 15 mL of isopropyl alcohol were added. The mixture was stirred at room temperature for 8 h and the reaction progress was monitored by TLC. The formed solid was filtered and washed with petroleum ether leading to 10.2 g of white solid, yield 80%, m.p.120 °C.

B. Ultrasound irradiation procedure:

In a round-bottom flask, 4-methylpyridine (0.051 mol, 5 mL) and 1-bromo-3-chloropropane (0.05 mmol, 5.08 mL) in 5mL of isopropyl alcohol were added. The reaction mixture was irradiated in an ultrasonic bath GT SONIC-P3/GT at 40kHz for 8 h and processed, as indicated in the classical procedure, obtaining 11 g, yield 86%.

C. Solvent free reaction procedure:

In a round-bottom flask, 4-methylpyridine (0.051 mol, 5 mL) and 1-bromo-3-chloropropane (0.05 mmol, 5.08 mL) were added, the mixture was stirred at room temperature for 8 h and processed, as indicated in the classical procedure, obtaining 6.4 g, yield 68%.

**Figure 11:**
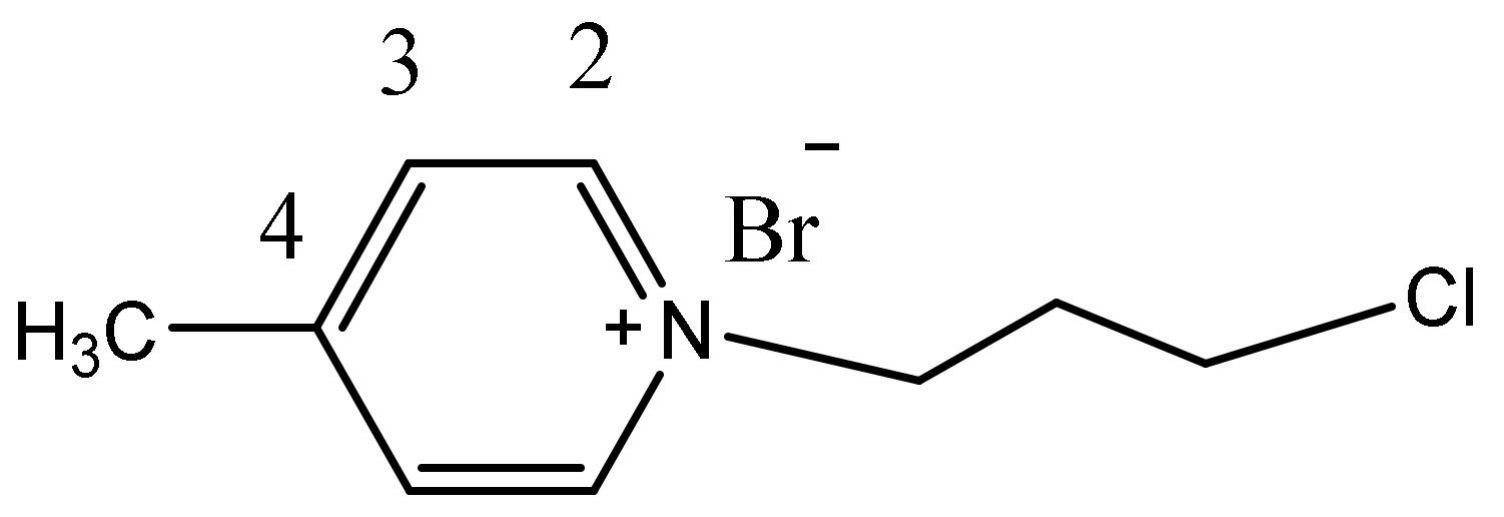
The structure of **1c** and the numbering of the atoms in phenyl ring.

**^1^H NMR** (CDCl_3_, 400 MHz) δ(ppm): 9.29 (d, 2H, J = 6.2 Hz, H^2^_Py_), 7.79 (d, 2H, J = 6.2 Hz, H^3^_Py_), 4.94 (t, 2H, J = 6.6 Hz, N^+^-CH_2_,), 3.56 (t, 2H, J = 6.6 Hz, Cl-CH_2_), 2.46-2.39 (m,2H, CH_2_), 2.46 (s, 3H, CH_3_), (see ESI S4). **^13^C NMR** (CDCl_3_, 100 MHz) δ(ppm): 159.3(qC^4^), 144.3(2C^2^), 128.7(2C^3^), 58.1 (N^+^CH_2_), 40.9(ClCH_2_), 33.8(CH_2_), 22.3 (CH_3_) (Figure 11). Anal. calcd. for: C_9_H_13_BrClN: C 43.14; H 5.23; N 5.59. Found: C 42.99; H 5.25; N 5.56.

7-bromo-10-ethyl-10H-phenothiazine-3-carbaldehyde **2a**

In a 100 mL bottom flask, 0.01 mol (2.5 g) of 3-formyl-10-ethylphenothiazine was dissolved in 25 mL of glacial acetic acid. To this mixture was added dropwise 0.0078 mol (1.25 g) of Br_2_, previously dissolved in 10 mL of glacial acetic acid. A reddish-brown precipitate was obtained that was stirred for 2 days, then water (100 mL) was added and the resulting yellow precipitate was filtered off. The obtained solution was stirred with 50 mL of toluene and the organic layer was separated, was dried over Na_2_SO_4_ and concentrated. The product was purified by column chromatography using silica gel. It was eluted with toluene to yield the product (1.77 g, 53%) as yellow solid, m.p. 95–97 °C (literature data 99–101 °C [19].

**Figure 12:**
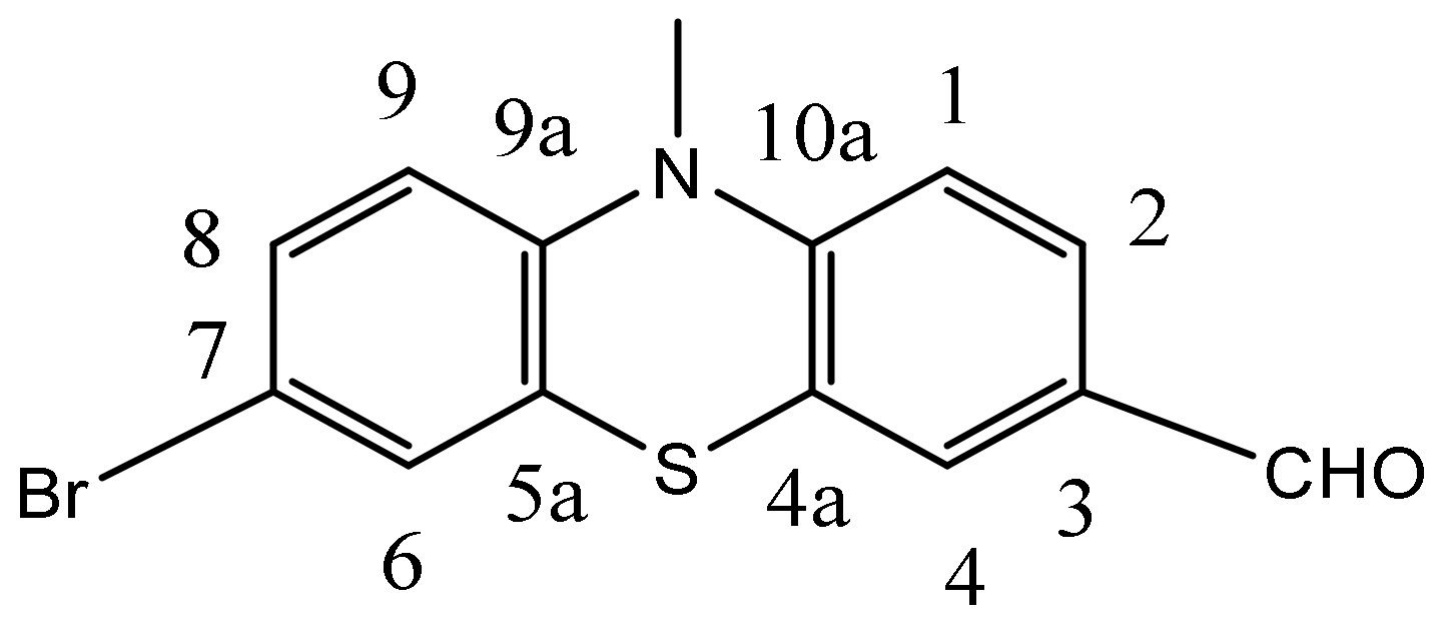
The structure of **2a** and the numbering of phenothiazine ring.

**^1^H NMR** (CDCl_3_, 400 MHz) *δ* (ppm): 9.78 (s, 1H, CHO), 7.62 (dd, 1H, J = 8.4 Hz, J = 1.9 Hz, H^2^), 7.53 (d, 1H, J = 1.9 Hz, H^4^), 7.23 (dd, 1H, J = 8.7 Hz, J = 2.2 Hz, H^8^), 7.18 (d, 1H, J = 2.2 Hz, H^6^), 6.88 (d, 1H, H_1_, ^3^J = 8.4, H^9^), 6.72 (d, 1H, J = 8.7 Hz, H^1^), 3.92 (q, 2H, CH_3_), 1.42 (t, 3H, CH_2_). **^13^C-NMR** (CDCl_3_, 100 MHz, δ(ppm): 189.8 (CHO), 149,7 (C^9a^) 142.2 (C^10a^), 131.1 (C^3^), 130.4 (C^6^), 130.2 (C^4^), 129.6 (C^8^), 128.1 (C^2^), 125.4 (qC^7^), 123.7(qC^5a^), 116.6(C^9^), 115.7(qC^4a^), 114.5 (C^1^), 42.5(CH_2_), 12.7(CH_3_), (Figure 12). HR-MS, (ESI; m/z): 

[M] ^+^ calcd. for C_15_H_12_BrNOS, 332.9817; Found 332.9807, (see ESI Figure S15).

(E)-4-(2-(7-bromo-10-ethyl-10*H*-phenothiazin-3-yl)vinyl)-1-methylpyridin-1-ium iodide **3b**

In a round-bottom vessel, 7-bromo-10-ethyl-10H-phenothiazine-3-carbaldehyde

(10 mmol, 3.3 g), 1,4-dimethylpyridin-1-ium iodide (11 mmol, 2.6 g) and catalytic amount of piperidine were added in 50 mL of isopropyl alcohol. The reaction mixture was stirred at room temperature for 3 h. The formed precipitate was filtered and successively recrystallized from ethanol, affording desired product 3.0 g as a dark red acicular crystal with 56% yield, m.p. = 250 °C.

**Figure 13:**
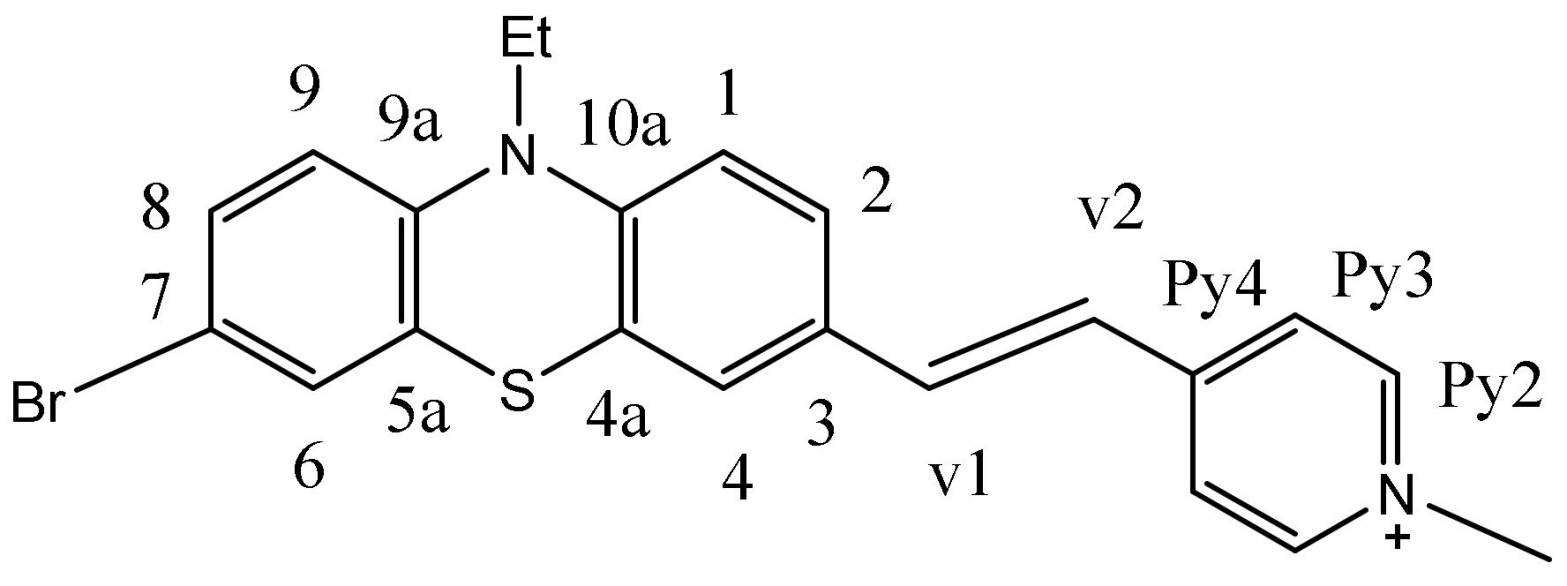
The structure of **3b** and the numbering of the atoms in the molecule.

**^1^H NMR** (DMSO-d_6_, 400 MHz) δ(ppm): 8.81 (d, 2H, J = 9.2Hz, H^2^_Py_), 8.13 (d, 2H, J = 6.5 Hz, H^2^_Py_), 8.13 (d, 2H, J = 6.5 Hz, H^3^_Py_); 7.90 (d, 1H, J = 16 Hz, H^2^_vinyl)_; 7.54-7.55 (m, 2H, H^2,4^_Ptz_); 7.40 (d, 1H, J = 16 Hz, H^1^_vinyl)_; 7.36-7.35 (m, 2H, H^8,6^_Ptz_), 7.11 (d, 1H, J = 9.0 Hz, H^9^_Ptz_), 6.98(d, 1H, J = Hz, H^1^_Ptz_) 4.23 (s, 3H, N^+^CH_3_); 3.94 (q, 2H, CH_2_); 1.29 (t, 3H, CH_3_), (see ESI S1, S2). **^13^C NMR** (DMSO-d_6_, 100 MHz) δ(ppm): 146.0(qC^4^_Py_), 145.5 (qC^9a^), 145.3(2C^2^_Py_), 143.0(qC^10a^), 139.8(qC^3^), 130.8(C^6^), 130.2(C^4^), 129.4 (C^8^), 129.3 (C^2^), 126.3 (C^2^vinyl), 124.8(C^1^vinyl), 123.5 (2C^3^_Py_), 122.8 (qC^7^), 121.8 (qC^5a^), 117.7 (qC^4a^), 116.1 (C^9^), 114.7 (C^1^), 47.2 (N^+^CH_3_), 42.0 (NCH_2_), 12.7 (CH_3_) (Figure 13). Anal. calcd. for: C_22_H_20_BrIN_2_S: C 47.93; H 3.66; N 5.08; S 5.82. Found: C 48.05; H 3.70; N 5.10; S 5.80.

(E)-1-(3-chloropropyl)-4-(2-(10-methyl-10H-phenothiazin-3-yl)vinyl)pyridin-1-ium bromide **3d**

A. Classical procedure:

In a round-bottom vessel, 10-methyl-10H-phenothiazine-3-carbaldehyde (10 mmol, 2.4 g), 1-(3-chloropropyl)-4-methylpyridin-1-ium bromide (11 mmol, 5.7 g) and catalytic amount of piperidine were added in 50 mL of isopropyl alcohol. The reaction mixture was stirred under reflux for 16h and the reaction progress was monitored by TLC. The reaction mixture was cooled, the precipitate was filtered and successively recrystallized from ethanol/acetonitrile (10:1, *v*/*v*) and isopropyl alcohol, affording desired product 3.1 g as a dark red solid with 70% yield, m.p. = 250 °C.

B. Ultrasound irradiation procedure:

In a pear-shaped flask, 10-methyl-10H-phenothiazine-3-carbaldehyde (1 mmol, 0.24 g), 1-(3-chloropropyl)-4-methylpyridin-1-ium bromide **7** (1.1 mmol, 0.57 g) and catalytic amount of piperidine were added in 15 mL of ethylic alcohol. The reaction mixture was irradiated in an ultrasonic bath GT SONIC-P3/GT at 40 kHz for 72 h. The reaction mixture was filtered and processed, as in the classical procedure above, obtaining 0.36 g, yield 80%.

C. Mechanochemical procedure:

In a mortar 10-methyl-10H-phenothiazine-3-carbaldehyde (1 mmol, 0.24 g), 1-(3-chloropropyl)-4-methylpyridin-1-ium bromide **7** (1.1 mmol, 0.57 g) and catalytic amount of piperidine were grinded with a pestle for 1h. The reaction mixture was successively recrystallized from ethanol/acetonitrile (10:1, *v*/*v*) and isopropyl alcohol, affording desired product 0.22 g (50% yield).

**^1^H NMR** (CDCl_3_, 600 MHz) δ(ppm): 8.94 (d, 2H, J = 5.7Hz, H^2^_Py_); 7.96 (d, 2H, J = 5.7 Hz, H^3^_Py_); 7.59 (d, 1H, J = 16 Hz, H^2^_vinyl)_; 7.34 (d, 1H, J = 7.6 Hz, H^2^_ptz_); 7.19 (s, 1H, H^4^_Ptz_); 7.01(t, 1H, J = 7.6 Hz, H^8^_ptz_); 6.93 (d, 1H, J = 16 Hz, H^1^_vinyl)_; 6.89 (d, 1H, J = 7.1 Hz, H^6^_Ptz_); 6.78(t, 1H, J = 7.1 Hz, H^7^_Ptz_); 6.63(d, 1H, J = 7.6 Hz, H^2^_Ptz_); 6.60 (d, 1H, J = 7.6 Hz, H^1^_Ptz_); 4.89 (t, 2H, N^+^CH_2_); 3.55(s, 3H, N-CH_3_); 3.50 (t, 2H, Cl-CH_2)_, 2.37-2.42(m, 2H, CH_2_), (see ESI S3a,b). **^13^C NMR** (CDCl_3_, 150 MHz) δ(ppm): 153.7 (qC^4^_Py_), 147.6 (qC^9a^), 143.9 (2C^2^_Py_),140.9 (qC^10a^), 140.0 (qC^3^), 129.1 (C^2^), 128.9(C^v2^), 127.7 (C^v1^), 126.9 (C^4^), 126.2(C^6^), 123.9 (2C^3^_Py_), 123.4(C^8^), 123.1(qC^4a^), 121.5 (qC^5a^), 120.1 (C^7^), 114.6 (C^9^), 114.3 (C^1^), 57.6 (N^+^CH_2_), 41.1 Cl-CH_2_), 33.7 (NCH_3_), 22.3 (CH_2_). Anal. calcd. for C_23_H_22_BrClN_2_S; C 58.30; H 4.68; N 5.91; S 6.77. Found: H 4.68; C 58.32; N 5.90; S 6.79.

(E)-1-methyl-4-(2-(10-methyl-10H-phenothiazin-3-yl)vinyl)pyridin-1-ium tetrafluoroborate **3a′**

To a solution of dye **3a** (9 mmol, 4.12 g) dissolved in 20 mL of MeCN, sodium tetrafluoroborate (10.9 mmol, 1.19 g) was added in a single portion. The reaction mixture was stirred at room temperature for 5 h and a small amount of reddish-brown precipitate was observed. The reaction mixture was concentrated by vacuum distillation, cooled and the solid residue was filtered and recrystallized repeatedly from MeCN to give a tetrafluoroborate salt in 85% yield (3.2 g) as a red-brown, microcrystalline solid m.p. 246 °C with decomposition.

The single crystal was obtained by slow evaporation of a solution formed by dissolving the compound **3a′** in an acetone:toluene mixture, 1:0.5 (*v*/*v*).

^1^H NMR (400 MHz, Acetone-d_6_, δ, ppm): 3.47 (s, 3H, N–CH_3_), 4.51 (s, 3H, N^+^CH_3_), 7.02–7.07 (m, 3H, H^1^, H^9^, H^7^), 7.18 (dd, 1H, *J* = 7.64 Hz, *J* = 1.5 Hz, H^6^), 7.26 (td, 1H, *J* = 7.68 Hz, *J* = 1.5 Hz, H^8^), 7.47 (d, 1H, *J* = 16.3 Hz, H^1^_vinyl_), 7.59 (d, 1H, J = 1.8Hz, H^4^), 7.63 (dd, 1H, J = 8.5Hz, J = 1.8Hz, H^2^), 7.99 (d, 1H, *J* = 16.3 Hz, H^2^_vinyl_), 8.27 (d, 2H, *J* = 6.7 Hz, H^3^_Py_), 8.92 (d, 2H, *J* = 6.7 Hz, H^2^_Py_). ^19^**F** NMR (376 MHz, Acetone-d_6_, *δ*, ppm): −152.8. Anal. calcd. for C_21_H_19_BF_4_N_2_S: C 60.30; H 4.58; N 6.70; S 7.67; Found: C 60.45; H 4.60; N 6.68; S 7.65.

### 3.3. X-ray Diffraction

Single crystal X-ray diffraction and refinement (SC)

A suitable single crystal of studied compound was placed on a fine nylon loop, was coated in inert oil (PARATONE N) and mounted on the goniometer of a SuperNova diffractometer. The diffractometer is equipped with dual micro-sources (Mo and Cu), EoS CCD detector and the tube operates at 50 kV and 0.8 mA. The diffraction intensities were collected at 120 K using CrysAlis PRO software [20]. The Lorentz, polarization and absorption factors were corrected as well in CrysAlis PRO [20].

The crystal structure of dye **3b** was solved by Direct Methods with SHELXS [21] solution program and refined by least squares minimization with SHELXL [22], which are implemented in Olex2 software [23]. Nonhydrogenoid atoms were localized in the Fourier difference map and were further refined anisotropically considering the displacement isotropic parameter Uiso(H) = 1.2Ueq(C) for all CH, CH_2_ groups and 1.5Ueq(C) for all CH_3_ groups. Hydrogen atoms were placed in idealized positions and treated as riding as follows: ternary CH refined with riding coordinates (C-H = 0.98 Å), secondary CH_2_ refined with riding coordinates (C-H = 0.97 Å), and idealized CH_3_ methyl groups refined as a rotating group (C-H = 0.96 Å). Detailed crystallographic and structure refinement details are given in Table 2.

All crystal packing and molecular drawings were generated using Mercury software [24].

The CIF file of dyes **3a′** and **3b** have been deposited with the Cambridge Crystallographic Data Centre, having the associated deposition numbers 2067603, respective 2024705. A copy can be obtained free of charge on written application to CCDC, 12 Union Road, Cambridge, CB2 1EZ, UK (Fax: +44-12-2333-6033); on request via e-mail to deposit@ccdc.cam.uk or by access to http://www.ccdc.cam.ac.uk (accessed on 12 March 2021).

### 3.4. Fluorescence Measurements

#### 3.4.1. One-Photon Excited Fluorescence on Solid Samples

One-photon excited time-resolved fluorescence measurements (OPE-FLIM) on solid samples were performed on a MicroTime200 time resolved confocal fluorescence microscope system (PicoQuant) coupled with an SR-163 spectrograph and equipped with a Newton 970 EMCCD camera (Andor Technology) previously described [25]. We used for excitation a laser diode, operating at 485 nm and the signal was filtered by a HQ519LP (Chroma, Foothill Ranch, CA, USA) emission filter. Images were obtained with a 60 × /NA = 1.2 water immersion objective. For the fluorescence spectra extracted from different spots in the solid sample deposited on microscope cover glass we used an integration time of 10 s, with the diode working in continuous mode.

#### 3.4.2. Fluorescence Quantum Yield

The calculation of quantum yield for **3a′** dye in solution was performed using Rhodamine-101 as standard and the parameters employed are summarized in Table 3.

The experimental procedure was performed according to Wϋrth et al. [26] with the mention that the samples were diluted due to the large difference in quantum yield between the standard used Rhodamine 101 [27] and **3a′**, and consequently the dilution factor was introduced in the Equation (1).

(1) $$\Phi x=\Phi \mathrm{st}\cdot \frac{A_{st}}{A_{x}}\cdot \frac{F_{x}}{F_{st}}\cdot \frac{n_{x}^{2}}{n_{st}^{2}}\cdot \frac{D_{x}}{D_{st}}$$

### 3.5. Cell Culture and Staining Protocol

Reagents used for cell viability studies: Dulbecco’s modified Eagle’s medium (DMEM), fetal bovine serum (FBS), glutamine, and antibiotics (penicillin/streptomycin mix) were purchased from Gibco (Carlsbad, CA, USA). Cell proliferation reagent WST-1 was bought from Roche Molecular Biochemicals (Indianapolis, IN, USA).

B16-F10 murine melanoma cell line was purchased from ATCC (American Tissue Cell Culture) being maintained in standard cultured conditions: 95% humidity, 5% of CO_2_ and 37 °C. Cell culture media was supplemented with 10% FBS, glutamine (2%) and antibiotics (penicillin/streptomycin mix, 1%). For viability evaluation, each dye in concentrations ranging from 0 to 15 µM was administered to B16-F10 cells, for 24 h.

For fluorescence imaging, 8 × 10^4^ cells/well, 2-well LabTek Chambered, were incubated with 13.7 µM PVP dye for 24 h, and kept at 37 °C in humidified atmosphere with 5% CO_2_. The next day, the cells were washed three times with phosphate buffer (1× PBS, pH 7.4) to remove the uninternalized dye, then were fixed by paraformaldehyde (4%) for 20 min at room temperature. A counterstaining with DAPI (0.5 µg/mL) was performed to see the localization of the dye comparing to the nucleus. Prior to and after this step the cells were washed with PBS 1×. Finally, the fixed B16-F10 cells were investigated through fluorescence microscopy as described in Section 3.7.

### 3.6. Viability Assay

Viability assay was performed using WST-1 cell proliferation reagent. WST-1 reagent (15 μL/well) was incubated for 30 min with treated B16-F10 cells, meanwhile being reduced to its dye form. Its absorbance was measured at 420 nm by a HT BioTek Synergy microplate reader (BioTek Instrument, Inc., Winooski, VT, USA). The results were expressed as survival percent with respect to control. Cells considered to be control, with 100% of viability, were incubated in DMEM only with WST-1.

### 3.7. Fluorescence Imaging

The conventional epi-fluorescence images of B16-F10 murine melanoma cells incubated with the synthesized PVP dyes **3a**–**d** were collected with an inverted Axio Observer Z1 Carl Zeiss microscope), employing a Compact Light Source HXP 120 C metal halide fluorescence light source. Concretely, the fluorescence was imaged with a G 365 excitation filter (i.e., filter set 49 from Carl Zeiss) for visualization of the cell nuclei stained with DAPI, and an excitation filter BP 470/40 (i.e., filter set 38 from Carl Zeiss) for localizing the synthesized dyes inside the cells. The fluorescence images were captured using a 63× iris oil immersion objective and a Zeiss AxioCam MRm monochrome microscope camera and then processed using the ZEN software.

Confocal one-photon excited Fluorescence Lifetime Imaging Microscopy (OPE-FLIM) assays on the B16-F10 murine melanoma cells post 24 h incubation with dyes **3a**–**d** were performed using the MicroTime200 system as described in Section 3.4. The signal was collected with a Plan N 40× objective with numerical aperture (NA) of 0.65 and filtered by a 50 µm pinhole. The laser power was set at 5 µW. For the fluorescence spectra extracted from the OPE-FLIM images, an integration time of 2 s was used.

Two-photon excited FLIM (TPE-FLIM) assays were performed using the previously described MicroTime200 time-resolved confocal fluorescence microscope system (from Pico-Quant) coupled to a Mira 900 Titanium: Sapphire tunable femtosecond laser (from Coherent), operating at 76 MHz. The incubated B16-F10 cells were imaged under the microscope under 800 nm laser beam excitation operating at 10 mW power, using the same system configuration described previously [28]. The signal was subsequently collected using a Plan N 40× objective with numerical aperture (NA) of 0.65 and spectrally filtered by a FF01-750 SP emission filter (Semrock, USA). The TPE-FLIM images were acquired and analyzed using the SymPhoTime 64 software provided by PicoQuant. TPE lifetime decay curves and time traces were recorded from different regions of interest in the TPE—FLIM images. OPE and TPE fluorescence spectra were extracted from FLIM images by using a SR-163 spectrograph equipped with a Newton 970EMCCD camera from Andor Technology coupled to an exit port of the main optical unit of MicroTime200 through a 50 µm optical fiber. The integration time used for the TPE fluorescence spectra of dye **3b** collected inside cells was 60 s, while for the fluorescence spectra of dye **3a′** recorded in solution we used an integration time of 2 s.

### 3.8. Lipophilicity Protocol

The lipophilicity protocol was adapted using previously reported methods [29,30].

(1) Solvent preparation (water saturated with octanol and octanol saturated with water): potassium phosphate aq buffer (0.1 M pH 7.0) and an equal volume of 1-octanol were agitated to allow saturation, then left to separate overnight.
(2) Preparation of dye stock solution: 100 μg / mL in DMSO.
(3) A total of 150 μL of dye stock solution was added to 1425 μL of water (V_w_) and the absorbance of this initial aqueous solution was measured (A).
(4) To the aqueous solution with the dye 1425 μL of 1-octanol (V_0_) was added.
(5) The sample was stirred for 2 h, then centrifuged (3500 rpm, 10 min) to separate the phases.
(6) The absorbance of the separate aqueous solution (A’) was measured.

The lipophilicity of dyes **3b** and **3c** were calculated according to the Equation (2) and the obtained values are presented in Table 4.

(2) $$\mathrm{logP}=\log\;(\frac{A-A\prime }{A\prime }\cdot \frac{Vw}{Vo})$$

## 4. Conclusions

To sum up, ultrasound irradiation is proposed as a convenient energy saving procedure for *N*-alkylation of pyridine derivatives as well as for Knoevenagel condensation of methyl-pyridinium salts with phenothiazine carbaldehydes, generating high yields (80–86%) of product in shorter reaction time as compared to convective heating or mechanochemical procedures.

The optical properties of the PVP dyes **3a**–**d** and **3a′**, described in this work are disclosing absorption maxima situated in 440–450 nm range in water which appeared red shifted to 495 nm when dissolved in low polarity organic solvents such as DCM and a good photostability in solution upon irradiation at 365 nm. Additionally, the steady-state and time-resolved emissive properties of dyes **3b** and **3a′** in solid state were assessed under one-photon excitation at 485 nm. Upon both conventional fluorescence microscopy (blue light excitation (450–490 nm)) and confocal imaging (OPE (485 nm) and TPE (800 nm)) PVP **3a**–**d** display fluorescence emission, making them suitable candidates for biological cell staining, as emphasized by fluorescence microscopy images collected from stained melanoma B16-F10 cells. All dyes were able to penetrate the plasma membrane and can bind differently with DNA from nuclei. In this context, we assume that the synthesized PVP dyes **3a**–**d** described here can be successfully used as fluorescent staining agents for in vitro imaging applications.

## Figures and Tables

**Table 1:** The structural characteristics, absorption and emission data recorded for PVP **3a**–**d** and **3a′**.

Cpd.	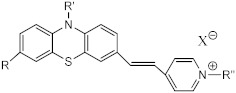				λ_abs_^a^	λ_em_^b^	Stokes Shift	
	R	R′	R″	X^−^	(nm)	(nm)	(cm^−1^)	Ref.
**3a**	H	–CH_3_	–CH_3_	I^−^	455	749	8627	[11]
**3a′**	H	–CH_3_	–CH_3_	BF4^−^	455	720 (605 *)	8089	
**3b**	Br	–C_2_H_5_	–CH_3_	I^−^	460	650	6354	
**3c**	H	–CH_3_	–C_4_H_9_	I^−^	458	669	6886	[11]
**3d**	H	–CH_3_	–(CH_2_)_3_-Cl	Br^−^	459	670	6861	

^a^ Conc. 10^−4^ M in DMSO solution; ^b^ **λ_em_** in solid state for dyes **3a**–**d** and **3a′**; * **λ_em_** in DMSO solution (Conc. 10^−^^8^ M) for **3a′**.

**Table 2:** Crystal data and structure refinement for dye **3b**.

Identification Code	Dye 3b
Empirical formula	C_22_H_20_BrIN_2_S
Formula weight	551.27
Temperature/K	123.1
Crystal system	Monoclinic
Space group	P2_1_/c
a/Å	17.6219(4)
b/Å	7.8451(2)
c/Å	15.0465(3)
α/°	90
β/°	101.306(2)
γ/°	90
Volume/Å^3^	2039.75(8)
Z	4
ρ_calc_g/cm^3^	1.795
μ/mm^−1^	15.650
F (000)	1080.0
Crystal size/mm^3^	0.1 × 0.15 × 0.17
Radiation	CuKα (λ = 1.54184)
2Θ range for data collection/°	10.238 to 141.238
Index ranges	−21 ≤ h ≤ 19, −9 ≤ k ≤ 5, −18 ≤ l ≤ 18
Reflections collected	7087
Independent reflections	3843 [R_int_ = 0.0299, R_sigma_ = 0.0447]
Data/restraints/parameters	3843/0/246
Goodness-of-fit on F^2^	1.046
Final R indexes [I > = 2σ (I)]	R_1_ = 0.0372, wR_2_ = 0.0961
Final R indexes [all data]	R_1_ = 0.0453, wR_2_ = 0.1033
Largest diff. peak/hole/e Å^−3^	1.11/−1.06

**Table 3:** Required parameters to calculate relative quantum yield for **3a′**.

Item	Samples	Expression	Calculated/ Reported Value
Quantum yield	Rhodamine 101 (Ethanol + 0.01% HCl)	Φ_st_	1 [26]
	**3a′** (Chloroform sol.)	Φ_x_	0.035
Absorbance at 525 nm	Rhodamine 101	A_st_	0.0265
	**3a′**	A_x_	0.07
Area of emission spectrum	Rhodamine 101	F_st_	19327.82
	**3a′**	F_x_	16255.18
Average refractive index value of solvent	Rhodamine 101	n_st_	1.85423
	**3a′**	n_x_	2.085
Dilution ratio	Rhodamine 101	D_st_	10^2^
	**3a′**	D_x_	10^3^

**Table 4:** Lipophilicity value for the dyes **3b–c**.

Dye	A	A′	Log P
**3b**	0.535	0.088	0.748
**3**c	0.453	0.080	0.711

## Data Availability

The data that support the findings of this study are available from the corresponding author upon reasonable request.

## Supplementary Materials

The following are available online at https://www.mdpi.com/1422-0067/22/6/2985/s1.
