# Supplementary material for: Novel (Phenothiazinyl)Vinyl-Pyridinium Dyes and Their Potential Applications as Cellular Staining Agents
Source: Int J Mol Sci. 2021 Mar 15;22(6):2985. doi: 10.3390/ijms22062985 (PMC7999001; doi:10.3390/ijms22062985)
Supplement: Supplementary file 1 [file ijms-22-02985-s001.pdf]

Electronic Supporting Information for:

"Novel (Phenothiazinyl)Vinyl-Pyridinium Dyes and Their Potential Applications as Cellular Staining Agents"

Bianca Stoean[1], Dumitrita Rugina[2], Monica Focsan[3], Ana-Maria Craciun[3], Madalina Nistor[2], Tamas Lovasz[1], Alexandru Turza[4], Dan Porumb[1], Gal Emese[1], Castelia Cristea[1], Luminita Silaghi-Dumitrescu[1], Simion Astilean[3,5], Luiza Gaina[1]*

[1] Research Center on Fundamental and Applied Heterochemistry, Faculty of Chemistry and Chemical Engineering, Babeş-Bolyai University, 11 Arany Janos str., RO-400028 Cluj-Napoca, România; ; sba2488@chem.ubbcluj.ro (B.S); tlovasz@chem.ubbcluj.ro (T.L.); pdan@chem.ubbcluj.ro (D.P.); emese@chem.ubbcluj.ro (E.G.); castelia.cristea@ubbcluj.ro (C.C.); lusi@chem.ubbcluj.ro (L.S.-D.);

[2]Biochemistry Department, Faculty of Veterinary Medicine, University of Agricultural Science and Veterinary Medicine, 3-5 Calea Manastur str., RO-400327 Cluj-Napoca, România; oliviapreda@gmail.com (D.R.); nistor.madalina@usamvcluj.ro (M.N.).

[3]Nanobiophotonics Laboratory, Institute for Interdisciplinary Experimental Research in Bionanoscience, Babeş-Bolyai University, 42 Laurian str., 400271 Cluj-Napoca, România; monica.iosin@phys.ubbcluj.ro (M.F.); ana.gabudean@ubbcluj.ro (A.M.C.);

[4] Department of Mass Spectrometry, Chromatography and Applied Physics, National Institute for Research and Development of Isotopic and Molecular Technologies, 67-103 Donat str., RO-400293 Cluj-Napoca, România; alexandru.turza@itim-cj.ro (A.T.);

[5] Biomolecular Physics Department, Faculty of Physics, Babeş-Bolyai University, 1 M. Kogalniceanu str., Cluj-Napoca 400084, România. simion.astilean@phys.ubbcluj.ro (S.A.)

* Correspondence: ioana.gaina@ubbcluj.ro (L.G.); Tel.: +40-264-593833

CONTENTS

^{1}H, AND COSY NMR SPECTRUM FOR 3b, 3d AND 1c	Figures S1-S4
X RAY DIFFRACTION DATA	Figures S5, S6
FLUORESCENCE EMMISON SPECTRA IN DIFERENT SOLVENTS FOR 3a'	Figure S7
TWO-PHOTON EXCITED FLUORESCENCE SPECTRUM OF DYE 3a' IN DMSO	Figure S8
UV-VIS ABSORPTION SPECTRA OF DYE 3b IN TRIS-EDTA BUFFER SOLUTION	Figure S9
PHOTOSTABILITY BEHAVIORS OF PVP 3b UNDER UV AT 365nm	Figure S10 and S11
UV-VIS AGREGATION BEHAVIOUR AT 40°C FOR DYE 3b	Figures S12
UV-VIS ABSORBTION MAXIMA FOR THE CATIONIC PVP DYE 3b AND 3d and 3a' IN DIFERENT SOLVENTS	Table S1
CALCULATED QUANTUM CHEMICAL PARAMETERS SUCH AS E, E_{HOMO}, E_{LUMO} AND LOGP AND PLOTS OF THE FRONTIER MOLECULAR ORBITALS FOR THE DYES 3a-d.	Tables S2 and S3
LIPOPHILICITY STUDIES	Figure S13 and S14
MS SPECTRA FOR 2a	Figure S15

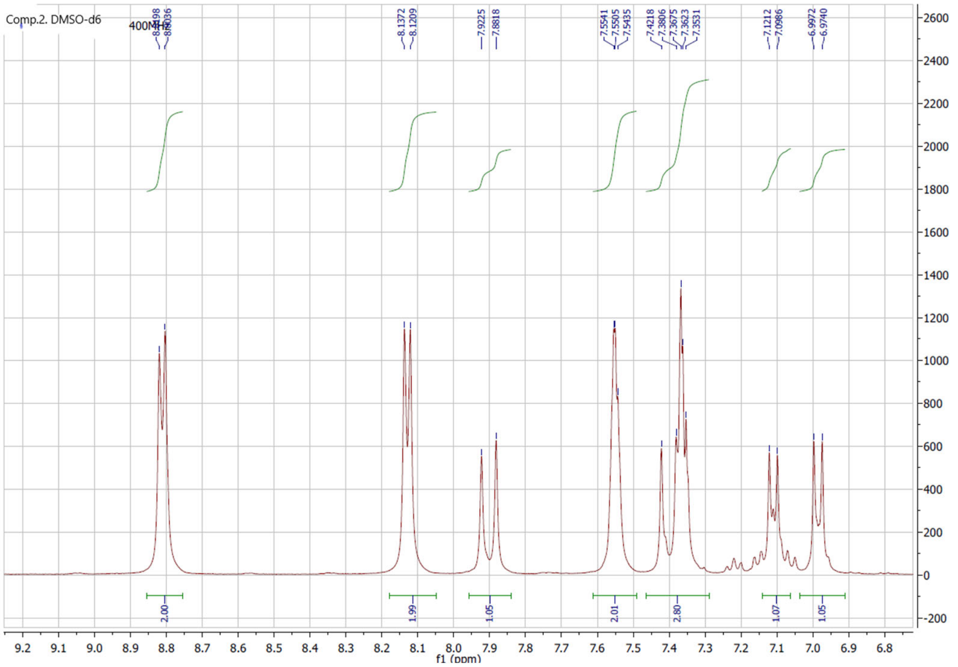

Fig. S1. ^{1}H-NMR spectra for compound **3b** in DMSO-d$_6$, 400MHz; aromatic region

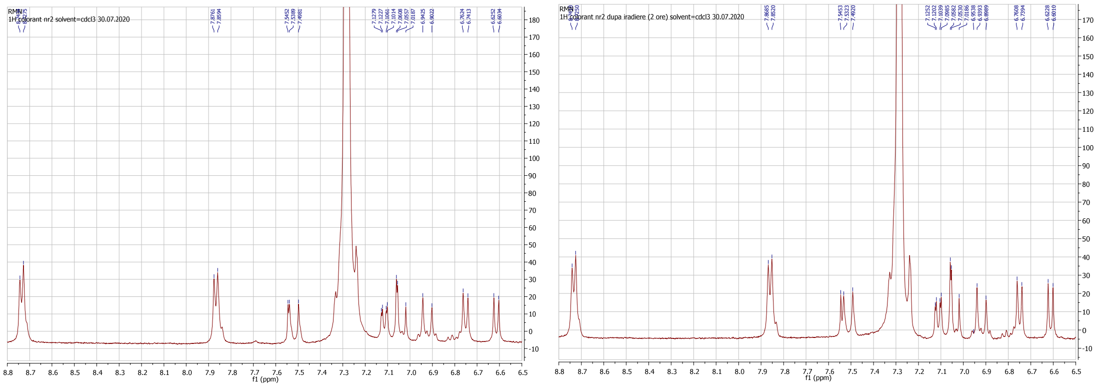

Fig. S2. ^{1}H-NMR spectra for compound **3b** in CDCl3, 400MHz, aromatic region; from left to right a). before irradiation; b). after 2h of irradiation at 365nm.

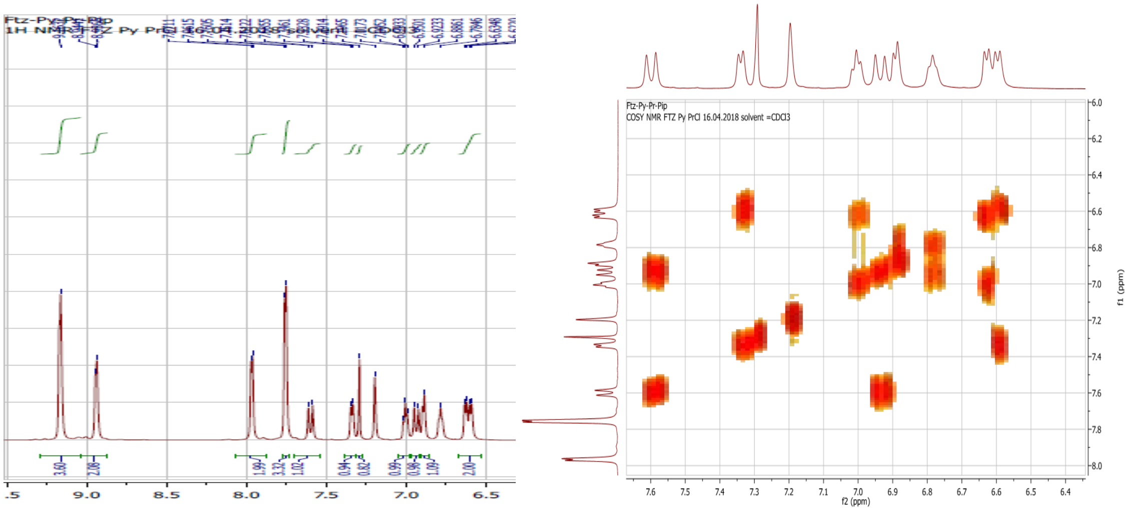

Fig. S3. a). ^{1}H-NMR spectra for compound **3d** in $CDCl_3$, compound **3d,** $CDCl_3$, at 600MHz; aromatic region; b) ^{1}H/^{1}H COSY spectra for **3d** aromatic region.

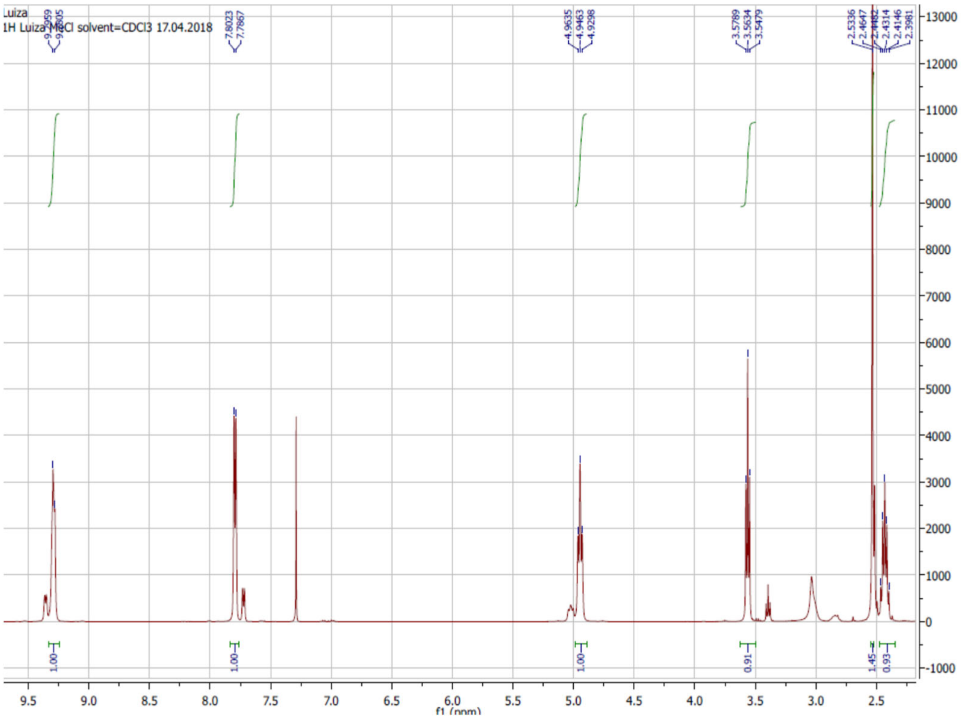

Fig. S4. ^{1}H-NMR spectra for the compound **1c** in $CDCl_3$, at 400MHz.

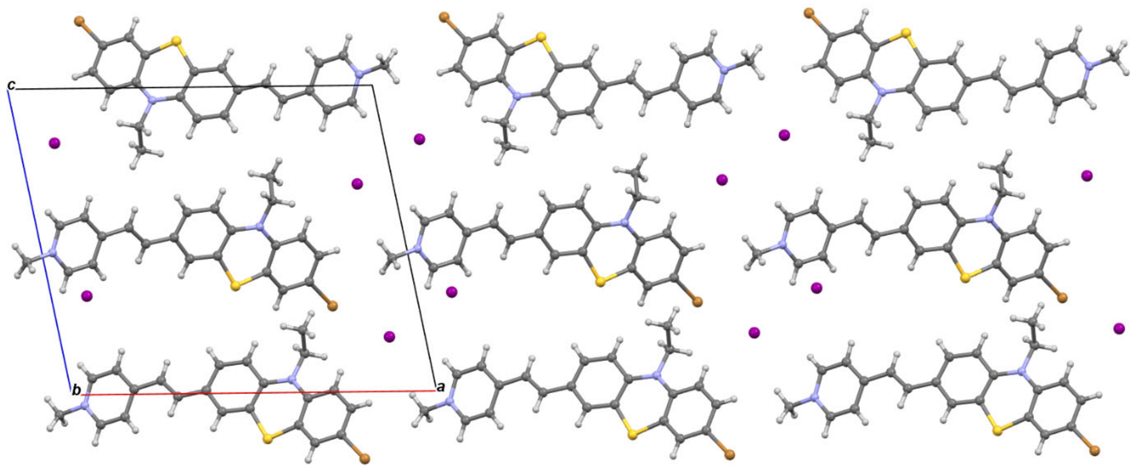

Fig. S5. View in the *aoc* plane highlighting the arrangement of the molecules in the *oa*-direction and the iodine ions in the space between them for **3b.**

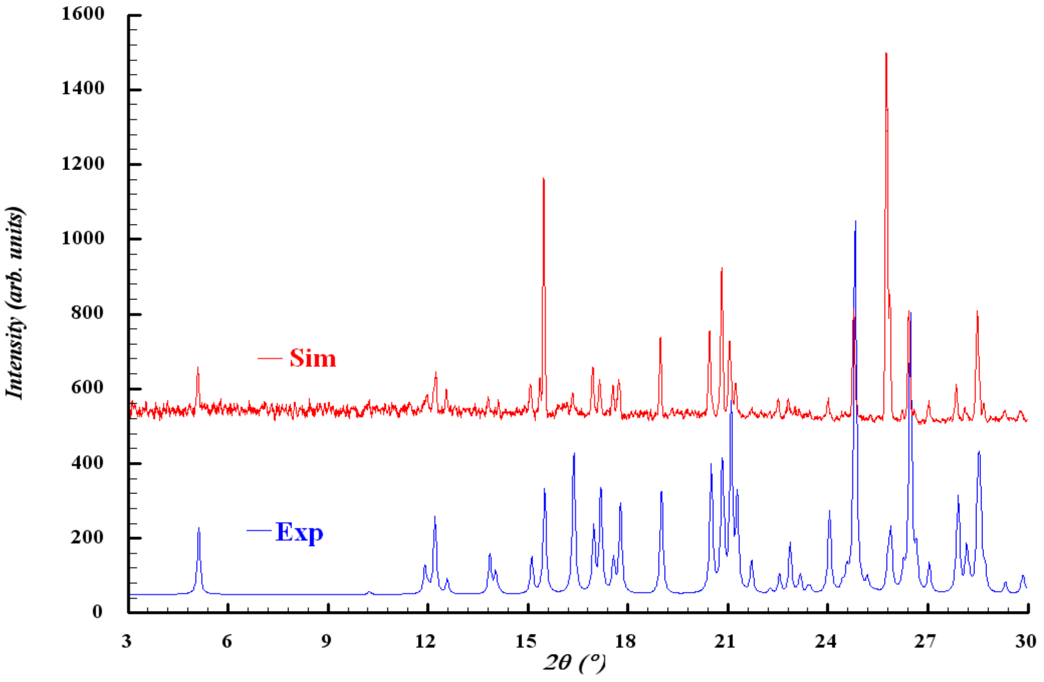

Fig. S6. Comparison between experimental and simulated XRD patterns for 3b powder.

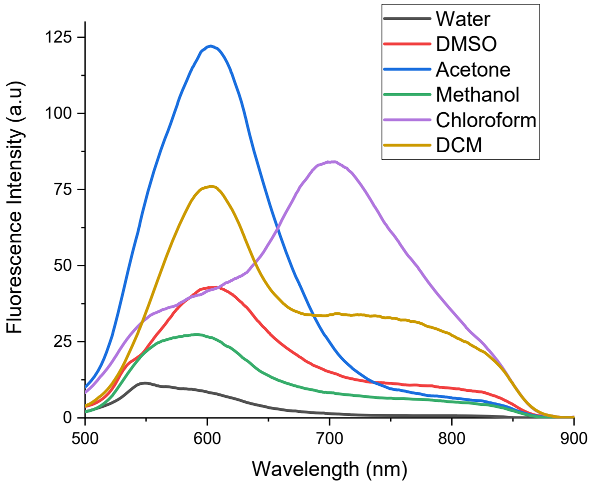

Fig. **S7**. Fluorescence emission spectra dye **3a'** (C_M = 3.38×10^{-8} M) in different solvents (λ_{exc}=460nm).

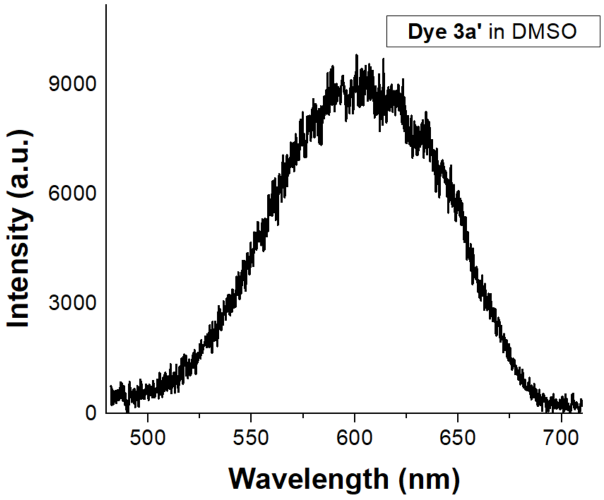

Fig. **S8**. Two-photon excited fluorescence spectrum of dye **3a’** in DMSO.

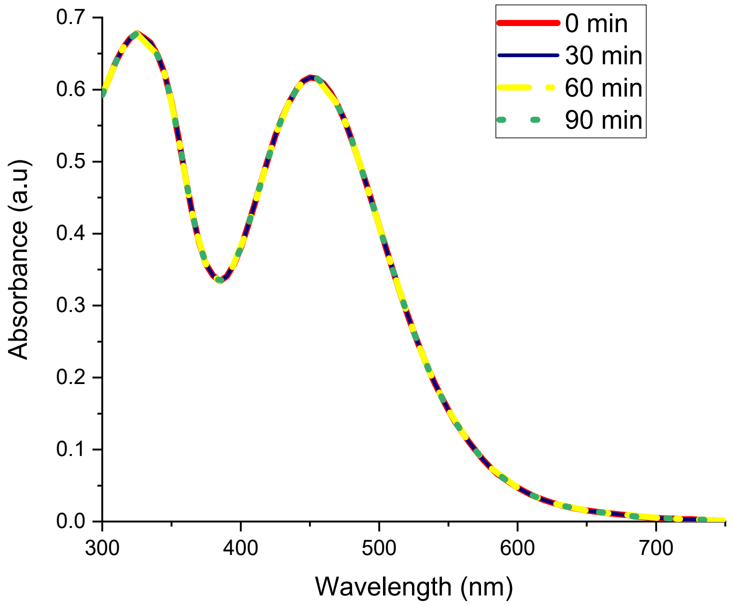

Fig. S9. UV-Vis absorption spectra of dye **3b** in TRIS-EDTA buffer solution (10^{-5} M) after 0, 30, 60, 90 min. irradiation with UV radiation λ=365 nm.

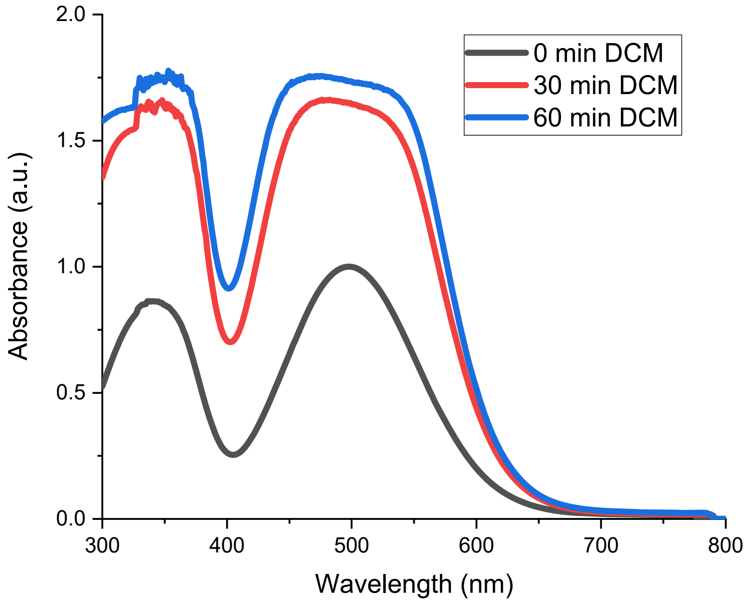

Fig. S10. Photostability of **3b** (0.033mM) in DCM under UV light irradiation (λ_{ex} = 365 nm)

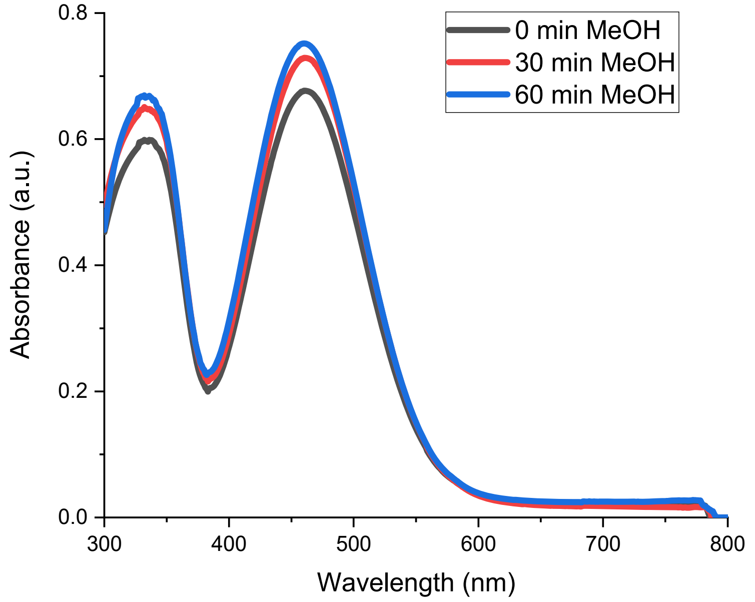

Fig. S11. Photostability of **3b** (0.33mM) in CH_3OH under UV light irradiation (λ_{ex} = 365 nm)

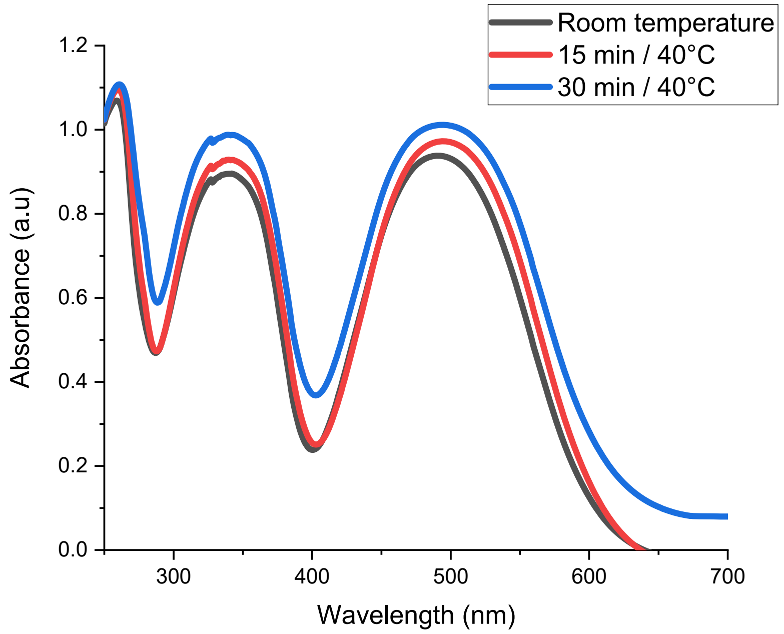

Fig. S12. UV-Vis absorption spectra of dye **3b** in chloroform solution (10^{-4} M) at room temperature and after heating at 40 ^{0}C for 15 and 30 min. respectively.

Table S1. The UV-Vis absorbtion maxima for the cationic PVP dyes 3a', 3b and 3d in different solvents

Comp.	$\lambda_{max,abs}$[nm][a]						$\lambda_{Emissie}$: [nm][b,c]
	H_2O	CH_3OH	DMSO	Acetone	CH_2Cl_2	$CHCl_3$	
3a'	435	451	455	454	491	497	587 [c]
3b	444	464	460	458	495	493	650 [b]
3d	450	466	459	458	480	496	670 [b]

[a] The maximum in the absorption spectrum. [b] In solid state excited at 450 nm. [c] In MeOH solution.

Table S2. Calculated quantum chemical parameters such as E, E_{HOMO}, E_{LUMO} and LogP for the dyes **3a-d** in different solvents

**Comp.**	**Conditions Solvent**	**E_{HOMO} [eV]**	**E_{LUMO} [eV]**	**E [au]**	**ΔE [eV]**	**Log P**
**3a**	Vacuum	-7.48	-5.60	-1319.16380	1.88	4.04
	Water	-5.24	-2.44	-1319.23421	2.80	
	Acetone	-5.21	-2.46	-1319.25337	2.75	
	DCM	-5.36	-2.71	-1319.24821	2.65	
	DMSO	-5.14	-2.34	-1319.25156	2.80	
**3b**	Vacuum	-7.48	-5.63	-3931.75416	1.85	5.21
	Water	-5.19	-2.47	-3931.82584	2.72	
	Acetone	-5.17	-2.48	-3931.84663	2.69	
	DCM	-5.33	-2.74	-3931.84177	2.59	
	DMSO	-5.10	-2.36	-3931.84429	2.74	
**3c**	Vacuum	-7.39	-5.50	-1437.11122	1.89	5.29
	Water	-5.23	-2.43	-1437.17781	2.80	
	Acetone	-5.20	-2.44	-1437.20008	2.76	
	DCM	-5.34	-2.70	-1437.19564	2.64	
	DMSO	-5.13	-2.33	-1437.19711	2.80	
**3d**	Vacuum	-7.48	-5.62	-1857.38527	1.86	5.03
	Water	-5.24	-2.47	-1857.45968	2.77	
	Acetone	-5.21	-2.48	-1857.48139	2.73	
	DCM	-5.36	-2.74	-1857.47628	2.62	
	DMSO	-5.14	-2.36	-1857.47885	2.78	

Table S3. Plots of the frontier molecular orbitals HOMO-LUMO in different solvents for dyes **3a-d** computed at DFT level of theory

**Comp.**	**Solvent**	HOMO	HOMO-1	LUMO	LUMO+1
**3a**	Vacuum	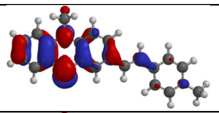	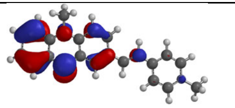	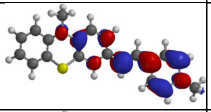	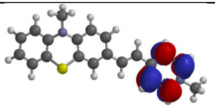
	Water	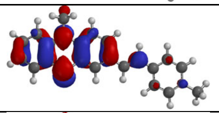	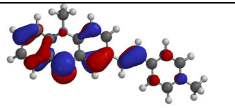	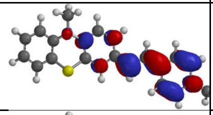	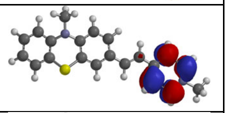
	Acetone	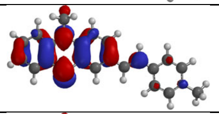	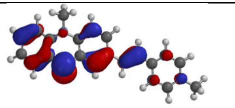	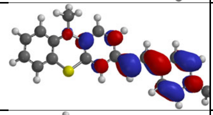	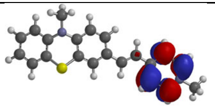
	DCM	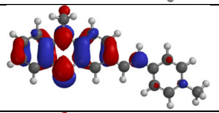	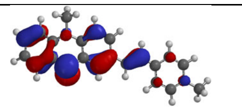	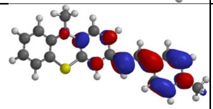	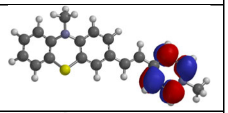
	DMSO	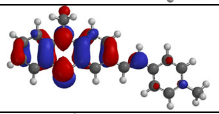	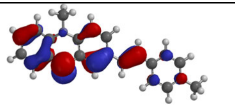	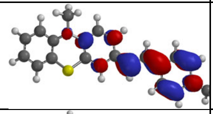	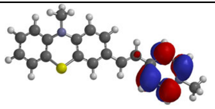
**3b**	Vacuum	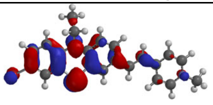	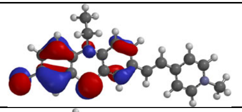	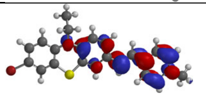	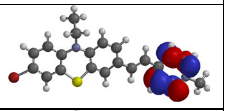
	Water	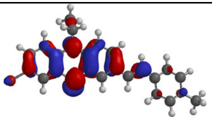	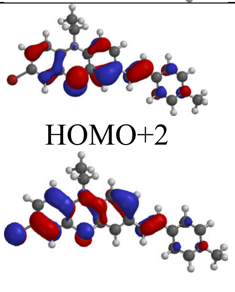 HOMO+2	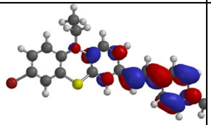	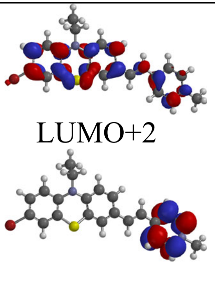 LUMO+2
	Acetone	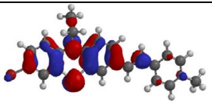	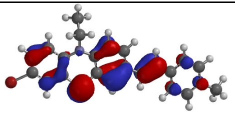	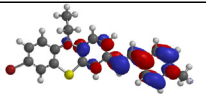	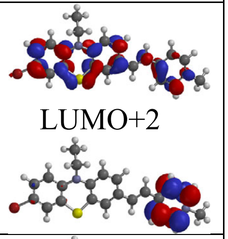 LUMO+2
	DCM	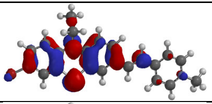	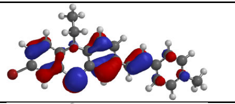	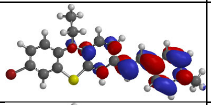	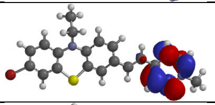
	DMSO	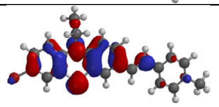	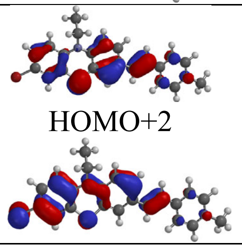 HOMO+2	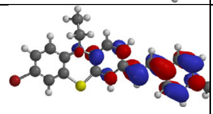	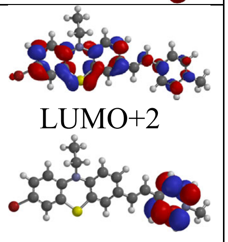 LUMO+2

**3c**	Vacuum	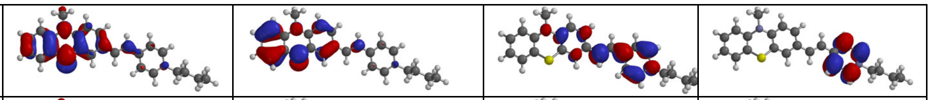			
	Water	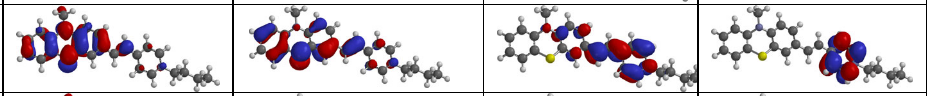			
	Acetone	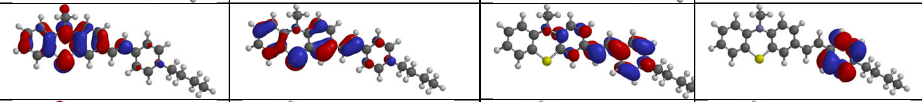			
	DCM	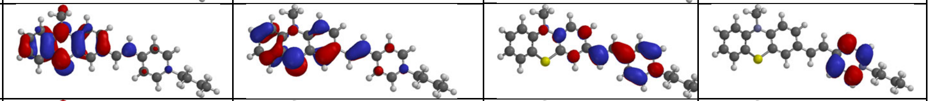			
	DMSO	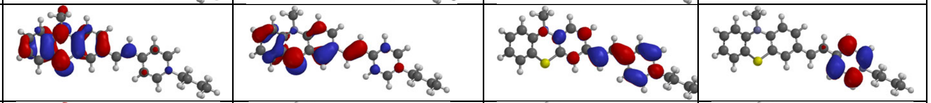			
**3d**	Vacuum	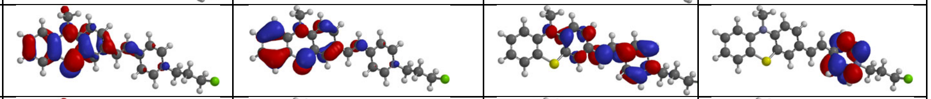			
	Water	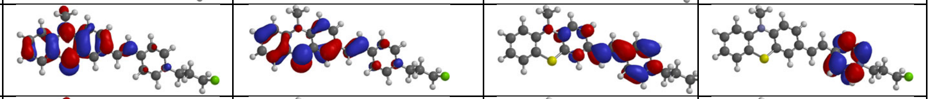			
	Acetone	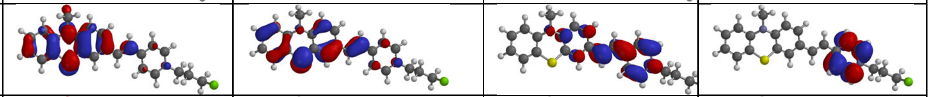			
	DCM	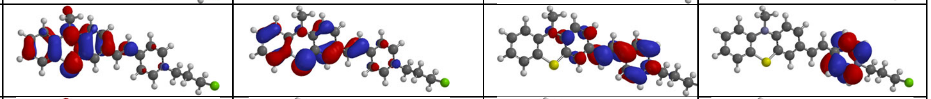			
	DMSO	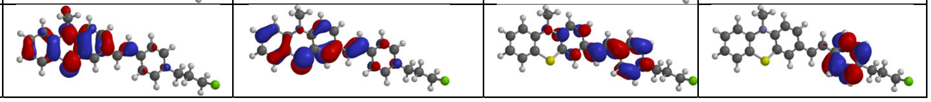			

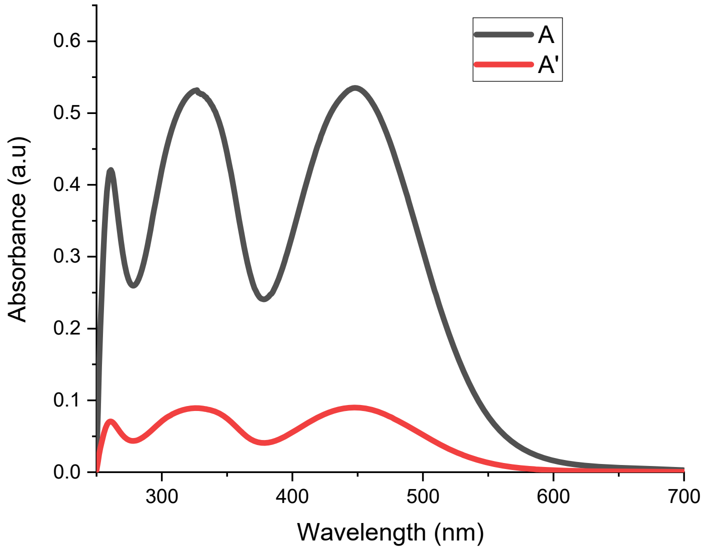

Fig.S13. UV-Vis absorption spectra for dye **3b** in aqueous solution: (A) before partition with octanol and (A') after partition with octanol

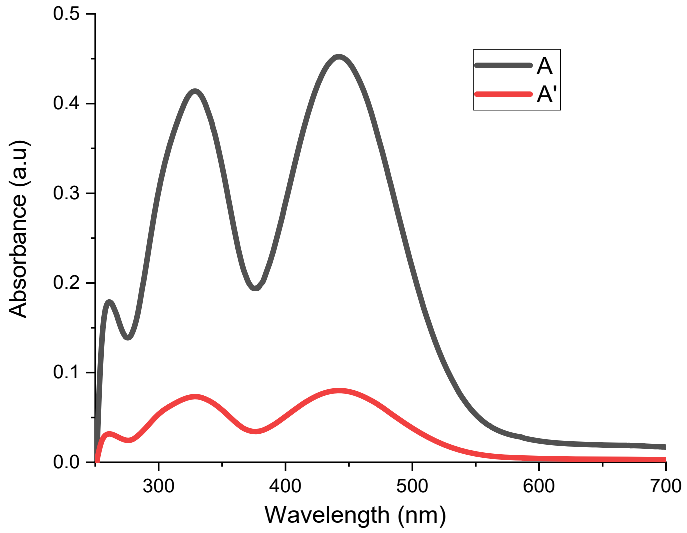

Fig.S14. UV-Vis absorption spectra for dye **3c** in aqueous solution: (A) before partition with octanol and (A') after partition with octanol

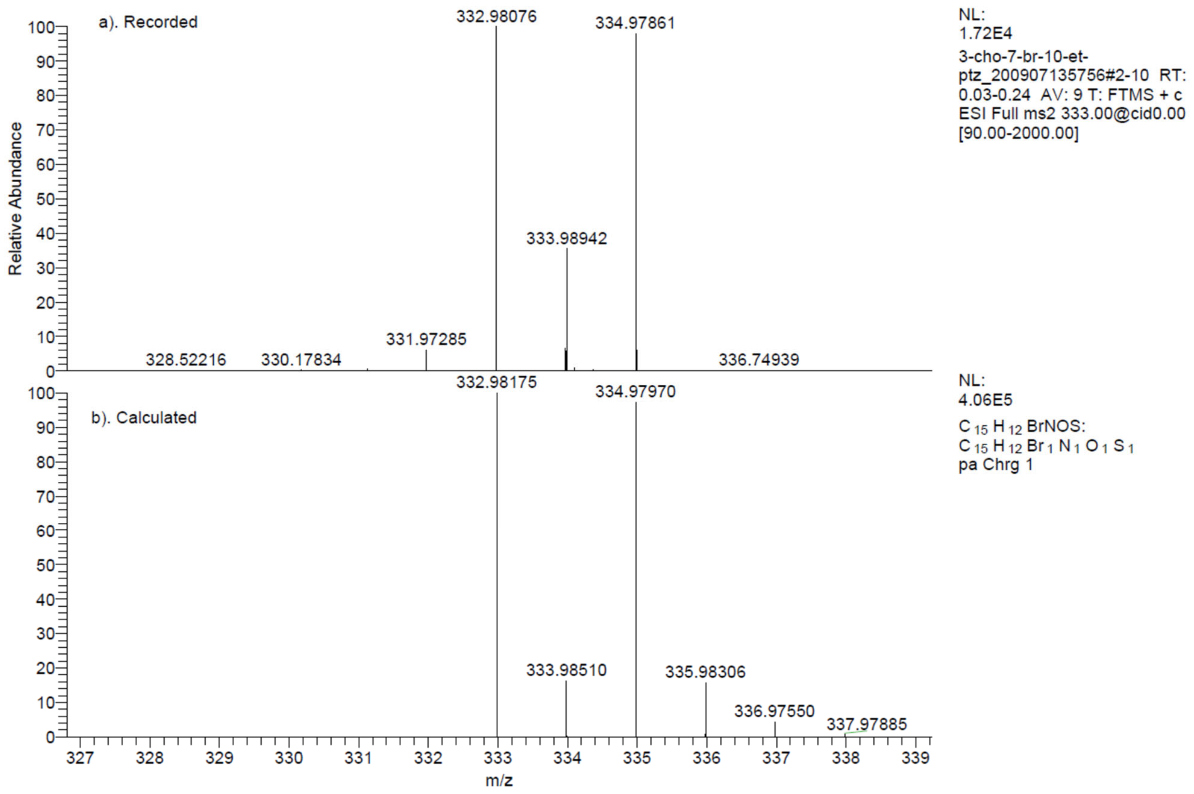

Fig. **S15**. HR-MS (ESI) spectra of **2a**.
